# Genome-wide enhancer RNA profiling adds molecular links between genetic variation and human cancers

**DOI:** 10.1186/s40779-024-00539-2

**Published:** 2024-06-11

**Authors:** Yi-Min Cai, Ze-Qun Lu, Bin Li, Jin-Yu Huang, Ming Zhang, Can Chen, Lin-Yun Fan, Qian-Ying Ma, Chun-Yi He, Shuo-Ni Chen, Yuan Jiang, Yan-Min Li, Cai-Bo Ning, Fu-Wei Zhang, Wen-Zhuo Wang, Yi-Zhuo Liu, Heng Zhang, Meng Jin, Xiao-Yang Wang, Jin-Xin Han, Zhen Xiong, Ming Cai, Chao-Qun Huang, Xiao-Jun Yang, Xu Zhu, Ying Zhu, Xiao-Ping Miao, Shao-Kai Zhang, Yong-Chang Wei, Jian-Bo Tian

**Affiliations:** 1 Department of Epidemiology and Biostatistics, School of Public Health, Research Center of Public Health, Renmin Hospital of Wuhan University, TaiKang Center for Life and Medical Sciences, Wuhan University, Wuhan, 430071 China; 2 Department of Gastrointestinal Oncology, Zhongnan Hospital of Wuhan University, Wuhan University, Wuhan, 430071 China; 3 Department of Cancer Epidemiology, Henan Engineering Research Center of Cancer Prevention and Control, Henan International Joint Laboratory of Cancer Prevention, the Affiliated Cancer Hospital of Zhengzhou University & Henan Cancer Hospital, Zhengzhou, 450008 China; 4 Department of Oncology, Tongji Hospital, Tongji Medical College, Huazhong University of Science and Technology, Wuhan, 430030 China; 5 Department of Gastrointestinal Surgery, Union Hospital, Tongji Medical College, Huazhong University of Science and Technology, Wuhan, 430022 China; 6 Department of Gastrointestinal Surgery, Zhongnan Hospital of Wuhan University, Wuhan University, Wuhan, 430071 China; 7 Department of Gastrointestinal Surgery, Renmin Hospital of Wuhan University, Wuhan, 430060 China; 8 Department of Epidemiology and Biostatistics, School of Public Health, Tongji Medical College, Huazhong University of Science and Technology, Wuhan, 430022 China; 9 Jiangsu Collaborative Innovation Center for Cancer Personalized Medicine, Nanjing Medical University, Nanjing, 211166 China; 10 Department of Gastrointestinal Oncology, Hubei Cancer Clinical Study Center, Zhongnan Hospital of Wuhan University, Wuhan, 430071 China

**Keywords:** Enhancer RNA, eRNA quantitative trait loci (eRNAQTLs), Genome-wide association study (GWAS), *ENSR00000155786*, *SENP7*

## Abstract

**Background:**

Dysregulation of enhancer transcription occurs in multiple cancers. Enhancer RNAs (eRNAs) are transcribed products from enhancers that play critical roles in transcriptional control. Characterizing the genetic basis of eRNA expression may elucidate the molecular mechanisms underlying cancers.

**Methods:**

Initially, a comprehensive analysis of eRNA quantitative trait loci (eRNAQTLs) was performed in The Cancer Genome Atlas (TCGA), and functional features were characterized using multi-omics data. To establish the first eRNAQTL profiles for colorectal cancer (CRC) in China, epigenomic data were used to define active enhancers, which were subsequently integrated with transcription and genotyping data from 154 paired CRC samples. Finally, large-scale case-control studies (34,585 cases and 69,544 controls) were conducted along with multipronged experiments to investigate the potential mechanisms by which candidate eRNAQTLs affect CRC risk.

**Results:**

A total of 300,112 eRNAQTLs were identified across 30 different cancer types, which exert their influence on eRNA transcription by modulating chromatin status, binding affinity to transcription factors and RNA-binding proteins. These eRNAQTLs were found to be significantly enriched in cancer risk loci, explaining a substantial proportion of cancer heritability. Additionally, tumor-specific eRNAQTLs exhibited high responsiveness to the development of cancer. Moreover, the target genes of these eRNAs were associated with dysregulated signaling pathways and immune cell infiltration in cancer, highlighting their potential as therapeutic targets. Furthermore, multiple ethnic population studies have confirmed that an eRNAQTL rs3094296-T variant decreases the risk of CRC in populations from China (*OR* = 0.91, 95%CI 0.88–0.95, *P* = 2.92 × 10^−7^) and Europe (*OR* = 0.92, 95%CI 0.88–0.95, *P* = 4.61 × 10^−6^). Mechanistically, rs3094296 had an allele-specific effect on the transcription of the eRNA *ENSR00000155786*, which functioned as a transcriptional activator promoting the expression of its target gene *SENP7*. These two genes synergistically suppressed tumor cell proliferation. Our curated list of variants, genes, and drugs has been made available in CancereRNAQTL (http://canernaqtl.whu.edu.cn/#/) to serve as an informative resource for advancing this field.

**Conclusion:**

Our findings underscore the significance of eRNAQTLs in transcriptional regulation and disease heritability, pinpointing the potential of eRNA-based therapeutic strategies in cancers.

**Supplementary Information:**

The online version contains supplementary material available at 10.1186/s40779-024-00539-2.

## Background

Large-scale genome-wide association studies (GWASs) have revealed thousands of genetic variants associated with human cancers, yet the molecular function of these variants remains an ongoing challenge [1]. The majority of variants from GWASs are located in non-coding regions, suggesting their potential role as transcriptional regulators influencing traits [2]. Quantitative trait locus (QTL) mapping for genetic variants linked to intermediate molecular traits, such as expression QTL (eQTL), could serve as invaluable tools to bridge the gap between genotypes and human phenotypes [3]. However, eQTL analysis explains a moderate fraction of GWAS results, so there is a need to explore genetic mechanisms from different perspectives.

Among the various factors that regulate transcription, enhancers play a pivotal role in initiating gene expression [4]. With advancements in global transcription assays, it has been demonstrated that active enhancers transcribe non-coding RNAs known as enhancer RNAs (eRNAs) [5, 6]. Emerging evidence suggests that eRNAs directly impact enhancers’ functionality rather than being mere by-products of their activity. These effects include regulating the chromatin accessibility of target promoters and subsequently recruiting RNA polymerase II [7, 8]. Additionally, eRNAs participate in the formation of long-range loops between enhancer and promoter, thereby reprogramming transcriptional regulation [9, 10]. Cap analysis of gene expression sequencing (CAGE-seq), a sensitive method used to detect eRNAs, has been systematically conducted by the Functional Annotation of the Mammalian Genome (FANTOM) consortium across approximately 400 human tissues and cell types [11]. However, limited access to biospecimens and available molecular assays hamper the application of CAGE-seq at a population level, making it challenging to detect low expression rates of enhancers. Conversely, RNA sequencing (RNA-seq) data serve as a convenient resource for quantifying eRNA [12], while large sample sizes aid in identifying clinically relevant eRNAs and factors governing their transcription. Therefore, a combination approach utilizing RNA-seq data to quantify active enhancers defined by the FANTOM and Encyclopedia of DNA Elements (ENCODE) has been proposed [13–15], enabling robust identification of highly expressed enhancers across diverse biological samples.

Given the fact that multiple enhancers can regulate the same gene and that eQTLs are depleted near critical genes with redundant enhancers, QTL analysis of eRNA expression is likely to provide additional insights into disease etiology [16]. Global fine-mapping for 21 autoimmune diseases revealed that approximately 60% of causal variants were mapped to enhancers producing eRNAs upon immune stimulation [17]. Recent research further demonstrated that a causal variant of lupus regulated the expression of interferon regulatory factor 8 by modulating eRNA expression and cell type-specific enhancer-promoter loop interactions [18]. Additionally, a robust set of enhancer regions expressing eRNAs in the human brain showed enrichment for genetic variants associated with autism spectrum disorders [19]. Despite a recent study analyzing the association between genetic variants and eRNA expression in multiple cancers, the characteristics and contributions of eRNAs to GWAS loci remain unexplored [20].

To address these gaps, a robust eRNA quantitative trait locus (eRNAQTL) analysis was performed to identify genetic variants that regulate eRNA transcription in 30 different types of cancers using data from The Cancer Genome Atlas (TCGA). Systematic integration of multi-omics data was employed to unravel the functional features of these eRNAQTLs and their related eRNAs. Furthermore, the first eRNAQTL map for a Chinese population with colorectal cancer (CRC) and adjacent normal tissues was established, elucidating the distinct role of eRNAQTLs in the genetic architecture underlying CRC. Through extensive investigations involving multiple ancestral populations and diverse multipronged experiments, it is aimed to uncover functional eRNAQTLs that influence CRC risk. Moreover, our findings will be presented in an easily accessible online database, providing a curated list of variants, genes, and drugs as a valuable resource for the scientific community. Overall, our study enhances the understanding of the molecular mechanisms by which genetic variations impact eRNA expression and ultimately contribute to tumorigenesis.

## Methods

### Integrative eRNAQTL analyses in 30 human cancers from TCGA

eRNA expression profiles were obtained from the eRic database (https://hanlab.uth.edu/eRic), which were generated by mapping RNA-seq reads to eRNA transcription regions and filtering out eRNAs with average expression values (reads per million, RPM) < 1. Furthermore, the following criteria were applied for filtering eRNAs: (1) eRNA region overlapped with known coding genes based on annotation from ENCODE (hg19); (2) eRNA expressed in less than 50% of samples. To minimize the effects of outliers on regression scores, the expression value for each eRNA across all samples was quantile normalized. Finally, a total of 4403 eRNAs were included for analysis (Additional file 1: Table S1).

The genotype data were downloaded from the TCGA database, which includes 898,620 single nucleotide polymorphisms (SNPs) detected by Affymetrix SNP Array 6.0. To increase the statistical power for eRNAQTL discovery, we conducted pre-phasing and imputation by IMPUTE2 (version 2.3.2, https://mathgen.stats.ox.ac.uk/impute/impute_v2.html#download) as described in our previous study [21]. Imputed genotypes underwent filtering based on the following criteria: (1) imputation confidence score (INFO) < 0.4; (2) minor allele frequency (MAF) < 5%; (3) SNP missing rate ≥ 5%; and (4) Hardy-Weinberg equilibrium *P*-value < 1 × 10^−6^. After imputation and quality control, an average of approximately 4,438,885 SNPs per cancer type remained for subsequent analyses.

The genome-wide cis-acting eRNAQTL analyses, which evaluate the associations between SNPs and normalized eRNA expression (1 Mb distance to the candidate SNP), were performed using a linear model implemented in FastQTL (version 2.0, http://fastqtl.sourceforge.net/). To account for potential confounding factors, adjustments were made for population structures, batch effects, and clinical status (age, sex, and tumor stage). Significance was defined as false discovery rate (FDR) < 0.05 calculated by Storey’s *q*-value method. Then, multi-omics data were used to comprehensively characterize the features of identified eRNAQTLs and their target eRNAs. Detailed procedures regarding these analyses are available in Additional file 2: Materials and Methods.

### Identification of genome-wide eRNAQTLs in Chinese CRC tissues

#### Study population

Assay for transposase-accessible chromatin with high throughput sequencing (ATAC-seq) and H3K27ac chromatin immunoprecipitation sequencing (ChIP-seq) were performed on 10 CRC patients, while RNA-seq was carried out in 154 CRC patients, including both primary tumor tissues and adjacent normal tissues. All participants were unrelated Han Chinese individuals who underwent surgical treatment at Zhongnan Hospital of Wuhan University and Tongji Hospital of Huazhong University of Science and Technology (Hubei Province, China), between January 2019 and February 2023. Peripheral blood samples and demographic characteristics were also collected at recruitment. Written informed consent for the usage of samples in the current study was obtained from all patients before surgery. The collection procedures of clinical specimens were approved by the hospitals responsible for sample collection, the Clinical Research Institution Review Committee and Ethics Review Committee of Wuhan University (WHU-LFMD-IRB2023003), as well as the Institutional Review Board of Tongji Medical College, Huazhong University of Science and Technology [2019 IEC (S319)].

#### Annotation of enhancers

Considering that chromatin accessibility and acetylation of H3K27 are commonly utilized for the identification of enhancer elements, as well as being predictive of the expression of nearby genes and enhancer activity in plasmid-based reporter assays [22], a combination of ATAC-seq and H3K27ac ChIP-seq signals was applied to annotate enhancers. In brief, peaks of ATAC-seq and H3K27ac ChIP-seq were called using MACS2 (version 2.2.9.1, https://pypi.org/project/MACS2/). To control for mappability, only peaks identified in at least two same kinds of tissues were retained. Subsequently, the overlaps between ATAC-seq and ChIP-seq datasets were determined using BEDtools (version 2.18, https://github.com/arq5x/bedtools2), with significant enhancers defined as peaks located outside the ± 2.5 kb flank region of transcriptional start sites.

#### Quantification of eRNA expression

According to the methodology described in a previously published study [14], potential eRNA-transcribing regions were defined as the ± 3 kb regions around the middle point of these annotated enhancers. To ensure quality control, annotation of protein-coding genes from ENCODE was downloaded, and any eRNA regions that overlapped with known coding genes (with 1 kb extension from both the transcriptional start site and transcriptional end site) were also excluded. Then, RNA-seq data were mapped to these eRNA regions and eRNA expression levels were normalized by reads per kilobase per million mapped reads (RPKM) for each eRNA in each sample. Those eRNAs expressed in more than 50% of samples with an average expression value (RPKM) ≥ 0.5 were defined as detectable eRNAs. The quality control and covariate adjustment pipeline used for mapping eRNAQTL in Chinese CRC followed a similar approach to that employed in TCGA.

### Study populations in three-stage GWASs

The multi-ethnic and multi-stage case-control studies consist of the initial genome-wide discovery stage, followed by two replication stages. After quality control filtering, 34,585 CRC cases and 69,544 healthy controls were included in the consortium. Detailed descriptions of the participating studies and demographic characteristics of the study subjects can be found in Additional file 1: Table S2 and Additional file 2: Materials and Methods. Briefly, the consortium included 27,515 Chinese participants and 76,614 European participants. The Chinese participants in the discovery stage were recruited from Beijing (Beijing-1, *n* = 2289) and Wuhan (Wuhan-1, *n* = 9180), while the replication I phase involved participants from Beijing (Beijing-2, *n* = 3046), and replication II phase involved participants from Wuhan (Wuhan-2, *n* = 13,000). As for European participants in the discovery stage, they were sourced from the Genetics and Epidemiology of Colorectal Cancer Consortium [23] (GECCO, *n* = 37,740), whereas replication I and II phases included individuals Prostate, Lung, Colorectal, and Ovarian (PLCO) Cancer Screening Trial [24] (*n* = 7398) and the UK Biobank [25] (UKB, *n* = 31,476). A summary of participating studies along with workflow details is provided in Additional file 2: Fig. S1. The study was approved by the Clinical Research Institution Review Committee and Ethics Review Committee of Wuhan University, the Institutional Review Board of Tongji Medical College, Huazhong University of Science and Technology, and the ethics committees of the hospitals involved. Written informed consent was obtained from all included participants during recruitment. Genotyping, quality-control procedures, imputation, and statistical analysis methods employed by these individual studies are described comprehensively in the Additional file 2: Materials and Methods.

### Cell lines and culture

The normal human intestinal epithelial cells HIEC-6 and CRC cell lines HCT116, HT15, and SW480 were obtained from the China Center for Type Culture Collection (Wuhan, China) in September 2021. They were cultured in Dulbecco’s Modified Eagle’s Medium supplemented with 10% fetal bovine serum (Gibco, USA) and 1% antibiotics (100 U/ml penicillin and 0.1 mg/ml streptomycin) at 37 °C in a humidified atmosphere containing 5% CO_2_. All cell lines used in this study were authenticated through short tandem repeat profiling (Applied Biosystems, USA), and they were tested to ensure the absence of mycoplasma contamination (MycoAlert, USA).

### Construction of plasmids and transfection

A total of 1000-bp DNA fragments containing SNP rs3094296-T or rs3094396-C allele were commercially synthesized and subcloned into the pGL3-promoter vector (Promega, USA) by Genewiz Biological Technology (Suzhou, China). Transfection of plasmids into the cells was carried out using Lipofectamine 3000 (Invitrogen, USA) according to the manufacturer’s protocol at a final DNA content of 100 ng per well in 96 well plates. For RNA interference, siRNA oligonucleotides targeting homeobox A5 (*HOXA5)*, sentrin-specific protease 7 (*SENP7*), SENP7-associated eRNA *ENSR00000155786*, and non-targeting siRNA control were purchased from Tsingke (Beijing, China) and transfected using Lipofectamine RNAiMAX (Invitrogen, USA). The sequences of siRNA are provided in Additional file 1: Table S3 and the knockdown effect was determined by quantitative reverse transcription PCR (qRT-PCR).

### Dual-luciferase reporter assay

Constructed luciferase vectors containing either the rs3094296-T or rs3094396-C allele and the pRL-SV40 Renilla luciferase plasmid (Promega, USA) were co-transfected into cells. The Dual-Luciferase Reporter Kit (Promega, USA) was utilized to perform the luciferase reporter assay according to the manufacturer’s recommendations. The luciferase activity was normalized by the luminescence value of Renilla luciferase relative to that of firefly luciferase.

### Chromatin isolation by RNA purification (ChIRP) assay

The ChIRP probe designer from online biosearch technologies was used to design antisense oligo probes specifically targeting eRNA *ENSR00000155786* (“odd” and “even”), with *LacZ* RNA probes serving as control. The probe oligos were synthesized with a 3’-Biotin-TEG modification and purified. ChIRP assays were performed using the BersinBio™ chromatin isolation by RNA purification Kit (Cat# Bes5104-1, BersinBio, China), following the manufacturer’s instructions. qPCR was conducted with retrieved DNA using the primers to check the enrichment of eRNA at specific genomic loci. The sequences of all ChIRP probes and ChIRP-PCR primers are listed in Additional file 1: Table S3.

### qRT-PCR

RNA was extracted from cells or tissues using TRIzol reagent (Thermo Fisher Scientific, USA). Subsequently, reverse transcription was performed using the SuperScript^®^ III First-Strand Synthesis System (Invitrogen, USA) with HiScript^®^ II Q Select RT SuperMix for qPCR (Vazyme, China). qRT-PCR analysis was conducted on QuantStudio 5 qPCR systems (Applied Biosystems, Thermo Fisher, USA) utilizing ChamQ SYBR qPCR Master Mix (Vazyme, China). The expression levels of the target gene were normalized to glyceraldehyde-3-phosphate dehydrogenase (GAPDH). All specific primers used in qPCR are listed in Additional file 1: Table S3. The *P*-values were calculated by a two-sided Student’s *t*-test.

### Western blotting assay

After 48 h of transfection, total protein was extracted from cells using radioimmunoprecipitation assay buffer (Beyotime, China) supplemented with protease inhibitors phenylmethanesulfonyl fluoride (Beyotime, China), cocktail (Sigma, USA), and PhosSTOP (Sigma, USA). Protein quantification was performed using a BCA reaction (Beyotime, China) and denatured at 99 °C for 5 min. An equal amount of protein was separated by electrophoresis on 10% SDS-PAGE gels and then transferred to 0.45 mm polyvinylidene difluoride membranes (Millipore, USA). The protein samples were incubated overnight at 4 °C with antibodies against SENP7 (1:1000, Cat# ER64927, HUABIO, China) and GAPDH (1:1000, Cat# ET1601-4, HUABIO, China). HRP-conjugated anti-rabbit IgG antibody (1:5000, Cat# SA00001-2, Proteintech, China) and HRP-conjugated anti-goat IgG antibody (1:5000, Cat# SA00001-4, Proteintech, China) was used as the secondary antibodies. Chemiluminescence signal detection was achieved using SuperSignal West Pico PLUS Chemiluminescent Substrate (Thermo Fisher Scientific, USA) by Image Lab software (version 6.1).

### Cell proliferation assay

Cells were seeded and transfected with siRNA in 24-well plates at a density of 5 × 10^4^ cells per well. After 36 h, cells were trypsinized and plated at a density of 2500 cells with a volume of 100 µl cell suspension in flat-bottomed 96-well plates. Following a specific incubation period, cell viability was measured using CCK-8 assays (Dojindo, Japan) according to the manufacturer’s suggestion.

### Statistical analysis

Differences in demographic characteristics between cases and controls were evaluated by Student’s *t*-test or Pearson *χ*^2^ test. For the association analysis, unconditional multivariable logistic regression analyses were adopted to estimate odds ratios (*OR*s) and 95% confidence intervals (CIs) for the relationship between SNPs and CRC risk while adjusting for gender, age, drinking, and smoking status. Multiple genetic models including allelic, recessive, and additive genetic models were applied to assess the risk of eRNAQTLs in CRC, respectively. For functional assays, data were expressed as mean ± SEM or mean ± SD. Statistical analyses were performed using two-sided independent Student’s *t*-test, paired two-sided Student’s *t*-test, and Spearman’s correlation analysis. All statistical analyses were performed by R (4.0.3) or PLINK (1.9) software. The threshold for statistical significance was *P* < 0.05.

## Results

### Systematic landscape of eRNAQTLs across 30 cancer types

**Fig. 1:**
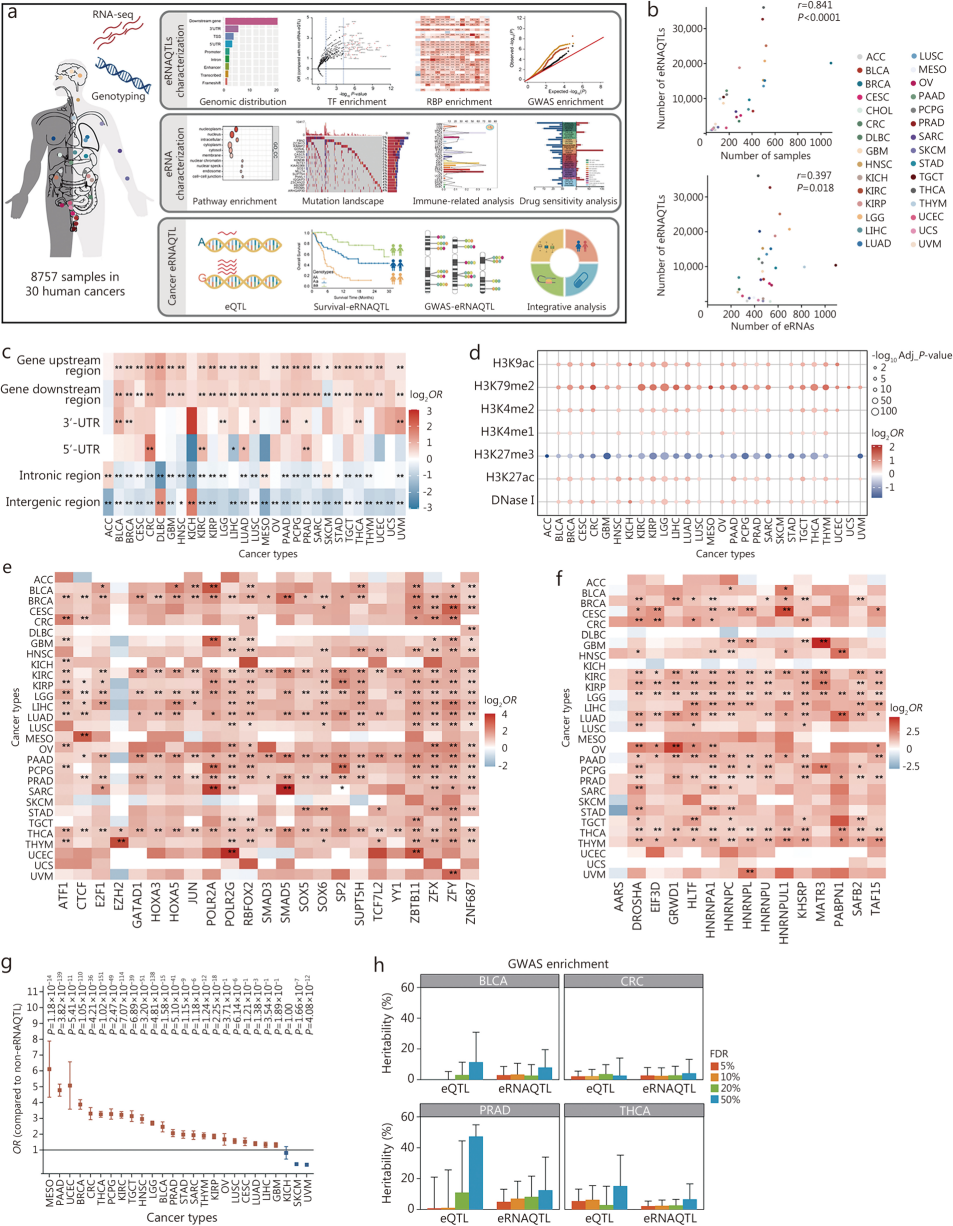
Systematic identification and functional characterization of eRNAQTLs across 30 human cancers. **a** Overview of eRNAQTL mapping in TCGA 30 cancer types. Firstly, RNA-seq reads mapped to enhancer regions and imputed genotypes from 8757 tumor samples were included to identify eRNAQTLs using the FastQTL algorithm. Subsequently, features of eRNAQTLs were characterized based on genomic distribution, functional annotation, and GWAS enrichment. Moreover, pathway analysis, mutation landscape, immune infiltration, and drug response were integrated to analyze the functions of target eRNAs in tumorigenesis and clinical application. Finally, to facilitate the exploration of eRNAQTLs by the wider research community, a database CancereRNAQTL was constructed, which includes three basic modules and extended modules. **b** Correlation between sample size, eRNA number after quality control, and identified eRNAQTLs number across multiple cancer types. **c** Enrichment of eRNAQTLs in different categories of genomic locations compared with control SNPs. Control SNPs are selected based on the matching number of variants in LD, MAF, and variant type. *P*-values were calculated by two-tailed Fisher’s exact test with Bonferroni correction according to the number of genomic regions analyzed. The color indicates log_2_ transformed *OR*, with red indicating *OR* > 1 and blue indicating *OR* < 1. **d** Bubble plot shows the enrichment of eRNAQTLs vs. random control SNPs in regulatory elements. The circle color represents log_2_ *OR*; the circle size represents the Bonferroni-corrected *P*-value considering the number of regulatory elements tested (7 elements). **e** Heatmap displays the enrichment of eRNAQTLs among variants within each TF binding site using ChIP-seq data from ENCODE, only the top significant TF in each cancer type was chosen for representation. *P*-values were calculated by two-tailed Fisher’s exact test with Bonferroni correction of the total number of TFs (that is 801). ^*^Bonferroni-corrected *P* < 0.05 and ^**^Bonferroni-corrected *P* < 0.01. The color indicates log_2_ *OR*, with red indicating *OR* > 1 and blue indicating *OR* < 1. **f** Enrichment of eRNAQTLs in individual RBPs using enhanced cross-linking immunoprecipitation sequencing (eCLIP-seq) data from ENCODE. *P*-values and log_2_ *OR* were calculated by two-tailed Fisher’s exact test after Bonferroni corrected the number of RBPs subjected to analyses (150 RBPs in total). **g** Enrichment of cancer-related GWAS signals by mapping eRNAQTLs to tag SNPs and their extended LD block. Results are calculated by two-tailed Fisher’s exact test with *P*-values corrected by the number of diseases tested. Error bars represent the 95% confidence intervals (CIs). **h** LD score regression partitioned heritability analysis for eQTLs and eRNAQTLs with varying FDR thresholds of 5%, 10%, 20%, and 50%. Error bars represent standard errors. ^*^P < 0.05, ^**^P < 0.01. TCGA The Cancer Genome Atlas, SNP single nucleotide polymorphism, LD linkage disequilibrium, MAF minor allele frequency, *OR* odds ratio, TF transcription factor, RBPs RNA binding proteins, ACC adrenocortical carcinoma, BLCA bladder urothelial carcinoma, BRCA breast invasive carcinoma, CESC cervical squamous cell carcinoma and endocervical adenocarcinoma, CHOL cholangiocarcinoma, CRC colon and rectum adenocarcinoma, DLBC lymphoid neoplasm diffuses large B-cell lymphoma, GBM glioblastoma multiforme, HNSC head and neck squamous cell carcinoma, KICH kidney chromophobe, KIRC kidney renal clear cell carcinoma, KIRP kidney renal papillary cell carcinoma, LGG lower grade glioma, LIHC liver hepatocellular carcinoma, LUAD lung adenocarcinoma, LUSC lung squamous cell carcinoma, MESO mesothelioma, OV ovarian serous cystadenocarcinoma, PAAD pancreatic adenocarcinoma, PCPG pheochromocytoma and paraganglioma, PRAD prostate adenocarcinoma, SARC sarcoma, SKCM skin cutaneous melanoma, STAD stomach adenocarcinoma, TGCT testicular germ cell tumors, THCA thyroid carcinoma, THYM thymoma, UCEC uterine corpus endometrial carcinoma, UCS uterine carcinosarcoma, UVM uveal melanoma, eRNAQTLs eRNA quantitative trait loci, RNA-seq RNA sequencing, ChIP-seq chromatin immunoprecipitation sequencing, ENCODE Encyclopedia of DNA Elements, GWAS genome-wide association study, eQTL expression quantitative trait locus, FDR false discovery rate

eRNAQTL analyses were initially performed based on the genotypes of germline variants, eRNA expression, and clinical parameters (age, sex, and tumor stage) of 8757 individuals across 30 cancer types (Fig. 1a; Additional file 2: Fig. S2). A total of 300,112 eRNAQTLs (FDR < 0.05) were identified, with a median of 10,004 eRNAQTLs per cancer type, ranging from 7 in cholangiocarcinoma (CHOL) to as many as 36,050 in thyroid carcinoma (THCA). Moreover, 802 survival-associated eRNAQTLs and 84,431 GWAS-associated eRNAQTLs were obtained (Additional file 1: Table S4). More eRNAQTLs were detected as sample sizes and the number of eRNA increases (Fig. 1b), implying that additional RNA-seq datasets and enhancer annotations may reveal more eRNAQTLs in the future.

The features of the identified eRNAQTLs were characterized by generating a set of matched control SNPs for comparison. According to the definition in SnpEff (version 5.0, https://pcingola.github.io/SnpEff/) [26], the position distributions of eRNAQTLs and non-eRNAQTLs were classified into 11 functional categories, including 3’ untranslated region (UTR), 5’ UTR, transcription factor (TF) binding site region, gene downstream region, gene upstream region, splice region, frameshift region, sequence feature region, non-coding exon region, intronic region and intergenic region (Additional file 2: Fig. S3a). Compared with control SNPs, eRNAQTLs exhibited significant enrichment in gene upstream and downstream regions, while being depleted in intergenic and intronic regions (Fig. 1c). Consistently, these eRNAQTLs were found to cluster within a proximity of 10 kb of H3K4me1 (enhancer marker) peaks center (Additional file 2: Fig. S3b). These results indicate that the identified eRNAQTLs are highly enriched in enhancer-related features.

### eRNAQTLs are enriched in genetic regulatory elements

Recently conducted studies have revealed that the initiation of eRNA transcription is regulated by chromatin accessibility and TFs [27, 28]. Therefore, we aimed to explore whether these eRNAQTLs could modulate eRNA expression by exerting control over epigenetic mechanisms. Using data from the ENCODE project, it was observed significant enrichment of eRNAQTLs in histone markers associated with enhancers (H3K4me1, H3K27ac) and active transcription (H3K4me2, H3K79me2, H3K9ac and DNase I hypersensitive sites), while there was a notable depletion in repressive transcription (H3K27me3; Fig. 1d; Additional file 1: Table S5). Further functional enrichment analysis of 801 transcriptional factor binding sites (TFBSs) identified 425 TFs that preferentially bound to regions surrounding eRNAQTLs (Fig. 1e; Additional file 1: Table S6). Notably, the CCCTC-binding factor (CTCF), a DNA binding factor involved in regulating 3D genome organization and transcriptional regulation, showed extensive enrichment [29]. Motif analysis also indicated the presence of CTCF within the region surrounding the eRNAQTL rs6481590-C allele in three cell lines (Additional file 2: Fig. S3c), indicating that different affinity for TFBSs may impact eRNA transcription. Additionally, among the tested set of 150 RNA binding proteins (RBPs), it was found that 73 RBPs exhibited significant enrichment (Fig. 1f; Additional file 1: Table S7), suggesting their potential roles in regulating eRNA transcription. These findings provide further insights into how epigenomic features contribute to the regulation of eRNA expression.

### eRNAQTLs provide an additional layer to functionally characterize cancer susceptibility

Emerging studies have demonstrated that the heritability of complex disease is disproportionately enriched in functional categories, particularly enhancers. To investigate whether our discovered eRNAQTLs can provide insight into the mechanisms underlying currently unexplained genome-wide associations, all cancer-related SNPs were first compiled from the GWAS catalog [30]. Subsequently, enrichment analyses were performed in the linkage disequilibrium (LD, *r*^2^ > 0.2) regions associated with these SNPs. Remarkably, compared to control SNPs, these eRNAQTLs were enriched in GWAS loci across 21 different types of cancer (Fig. 1g).

Considering that eQTLs only explained a moderate fraction of GWAS signals [2, 3] and that eRNA signals may better explain quantitative traits because of tissue or cell-type specificity of enhancers, we further evaluated whether eRNAQTLs could serve as a supplement to disease heritability estimation. Partitioned heritability estimation based on LD score regression showed that eRNAQTLs accounted for approximately 2 to 5% of the overall heritability in CRC, which was comparable to that explained by eQTLs. Interestingly, for bladder urothelial carcinoma (BLCA) and prostate adenocarcinoma (PRAD), eRNAQTLs contributed more significantly to heritability than their corresponding eQTLs. However, eRNAQTLs (2.2%, FDR < 0.05) contributed less to heritability than eQTLs (5.4%, FDR < 0.05; Fig. 1h) in thyroid carcinoma (THCA). These findings suggest that exploring molecular mechanisms through studying eRNAQTLs might provide additional insights into disease vulnerability.

### eRNAs facilitate the elucidating of the biological process of cancers

**Fig. 2:**
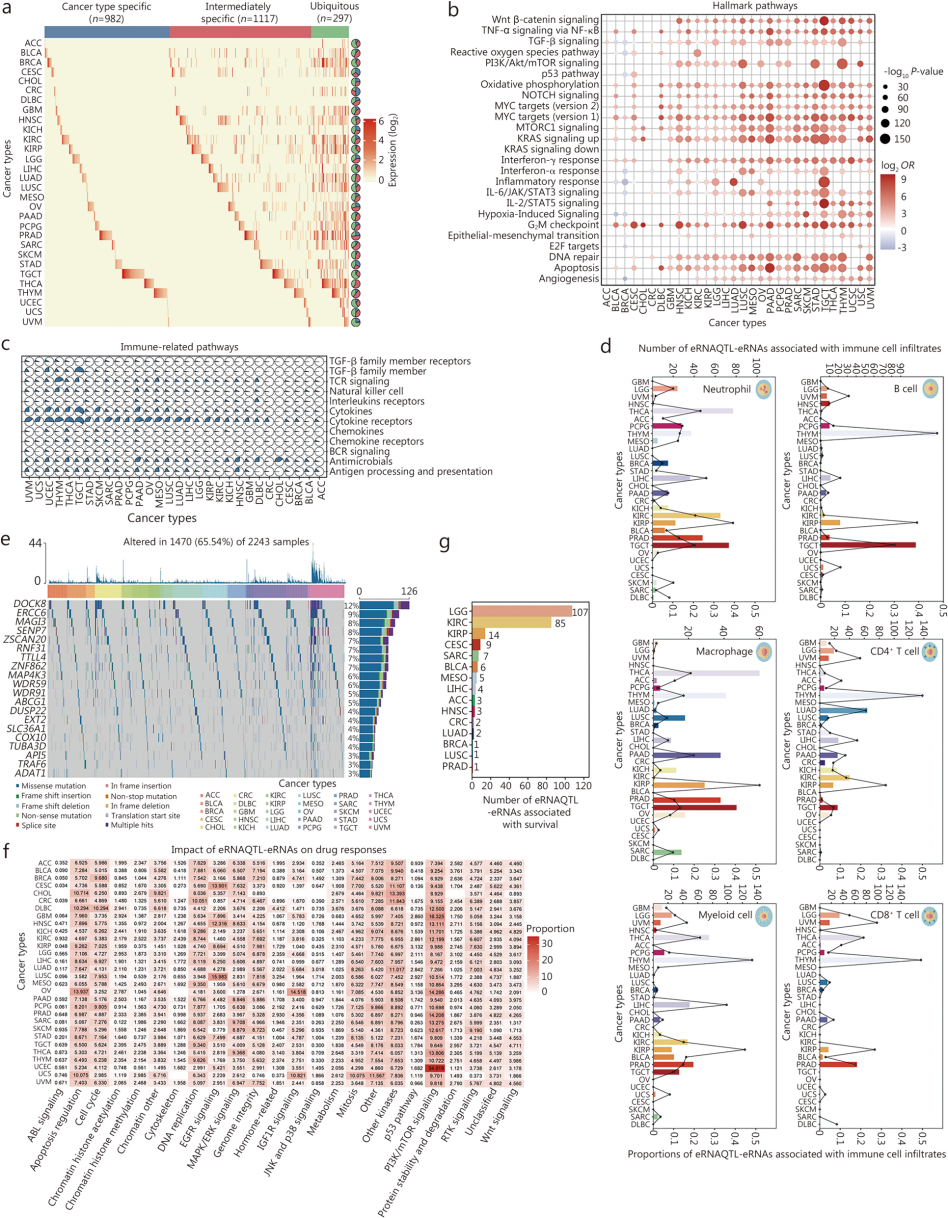
eRNAQTL-eRNAs play significant roles in tumorigenesis and clinical utility. **a** Expression profile of eRNAQTL-eRNAs in human cancers. Blue, red, and green bars denote cancer type-specific, intermediately specific, and ubiquitous eRNAs, respectively. Pie charts reflect the percentage of eRNAQTL-eRNAs in each category. **b** Enrichment analysis of the number of eRNAQTL-eRNAs in 50 hallmark gene sets by GSEA based on the rank score of each eRNA and gene. **c** Scatter pie plot of the proportion of eRNAQTL-eRNAs enriched in 17 immune-related pathways based on GSEA. **d** The number of eRNAQTL-eRNAs associated with immune cell infiltration estimated by TIMER in each cancer type (top Y-axis). The bottom Y-axis shows the proportion of these immune-related eRNAQTL-eRNAs. **e** Mutation landscape for target genes of eRNAQTL-eRNAs among 2243 TCGA samples. The top panel shows individual tumor mutation rates while the middle panel details cancer types for each patient. The bottom panel shows genes with relatively high mutation frequency in multiple cancer types. Mutation types are indicated in the legend at the bottom. **f** Proportion of eRNAQTL-eRNAs associated with drug response from GDSC dataset among different cancer signaling pathways. **g** Number of prognostic eRNAQTL-eRNAs in different cancer types. eRNAQTL eRNA quantitative trait locus, GSEA gene set enrichment analysis, TIMER Tumor Immune Estimation Resource, GDSC Genomics of Drug Sensitivity in Cancer, ACC adrenocortical carcinoma, BLCA bladder urothelial carcinoma, BRCA breast invasive carcinoma, CESC cervical squamous cell carcinoma and endocervical adenocarcinoma, CHOL cholangiocarcinoma, CRC colon and rectum adenocarcinoma, DLBC lymphoid neoplasm diffuses large B-cell lymphoma, GBM glioblastoma multiforme, HNSC head and neck squamous cell carcinoma, KICH kidney chromophobe, KIRC kidney renal clear cell carcinoma, KIRP kidney renal papillary cell carcinoma, LGG lower grade glioma, LIHC liver hepatocellular carcinoma, LUAD lung adenocarcinoma, LUSC lung squamous cell carcinoma, MESO mesothelioma, OV ovarian serous cystadenocarcinoma, PAAD pancreatic adenocarcinoma, PCPG pheochromocytoma and paraganglioma, PRAD prostate adenocarcinoma, SARC sarcoma, SKCM skin cutaneous melanoma, STAD stomach adenocarcinoma, TGCT testicular germ cell tumors, THCA thyroid carcinoma, THYM thymoma, UCEC uterine corpus endometrial carcinoma, UCS uterine carcinosarcoma, UVM uveal melanoma, WNT wingless-related integration site, RTK receptor tyrosine kinase, PI3K/mTOR phosphoinositide 3-kinase/mammalian target of rapamycin pathway, JNK and p38 Jun N-terminal kinase and p38 mitogen-activated protein kinase, IGF1R insulin-like growth factor 1 receptor, MAPK/ERK mitogen-activated protein kinase/extracellular signal-regulated kinase, EGFR epidermal growth factor receptor, ABL abelson

To further elucidate the functional roles of eRNAs regulated by eRNAQTLs (eRNAQTL-eRNAs) in human cancers, we initially examined their expression patterns. Interestingly, a total of 982 (40.98%, *n* = 2396) eRNAs were expressed exclusively in one cancer type, with testicular germ cell tumors (TGCT) having the highest proportion of cancer-type-specific eRNAs. It was noted that 1117 (46.62%, *n* = 2396) eRNAs showed intermediate specificity and were expressed in 2–9 different cancer types, while 297 (12.40%, *n* = 2396) eRNAs were ubiquitously expressed across ≥ 10 cancer types (Fig. 2a; Additional file 1: Table S8). These results highlight the lineage-specific generation of eRNAs in distinct cancer types, which may offer valuable insights into their physiological function.

Based on the current understanding that enhancers must ultimately converge on their target genes, it is crucial to establish a link between eRNAs and their downstream genes to dissect eRNA function [31]. The prediction of target genes for eRNAs was achieved by integrating physical distance (≤ 1 Mb) and co-expression analyses (the absolute value of partial correlation coefficient ≥ 0.3 and FDR < 0.05). This resulted in a total of 14,670 interactions involving 6551 putative target genes and 2132 eRNAs (Additional file 1: Table S9). Furthermore, Gene Ontology (GO) [32] and Kyoto Encyclopedia of Genes and Genomes (KEGG) [33] pathway analyses revealed that these target genes were predominantly associated with transcriptional regulation and cancer immune-related pathways (Additional file 2: Fig. S4). To investigate the specific pathways in which these target genes are involved, 50 hallmark gene sets and 17 immunologically relevant gene sets were collected to conduct gene set enrichment analysis (GSEA) [34]. In samples from more than 20 cancer types, the genes regulated by eRNAs were found to be involved in canonical cancer pathways, including MYC, KRAS proto-oncogene (KRAS), and DNA repair pathways (Fig. 2b). Moreover, an average of 27.49% of these genes showed activity in the cytokine receptor pathway, which is regarded as a potential target for immunotherapy treatment (Fig. 2c). These results demonstrate that eRNAs play an extensive role in the regulation of oncogenes as well as cellular responses to external signals like hypoxia or inflammation.

Given that immune responses to cancer are represented by diverse immune/inflammatory cell infiltrates, evaluating the factors influencing these immune infiltrates may provide novel insight into the development of cancers. We subsequently estimated the abundance of tumor-infiltrating immune cells using the Tumor Immune Estimation Resource (TIMER) [35], and identified 6 distinct subsets. Correlation analyses unveiled a median of 100 eRNA regulators associated with immune infiltration in each type of cancer (Fig. 2d; Additional file 1: Table S10), providing a valuable resource for determining the underlying mechanisms of the immune regulation of cancer. Overall, the identification of the targets of eRNAs enhances our comprehension of their functions and sheds light on how enhancer activation contributes to tumor progression.

### The potential clinical relevance of eRNAs

The uncontrolled proliferation of cells and the promotion of tumor-related inflammation are two hallmarks of cancer that facilitate the acquisition of genomic alterations by cancer cells, leading to genome instability [36]. Therefore, we explored the attributes of eRNA target genes in relation to cancer genomic events and found that these genes, such as dedicator of cytokinesis 8 (*DOCK8*) and excision repair 6 (*ERCC6*), exhibit a high burden of mutations and amplifications or deletions change (Fig. 2e; Additional file 2: Fig. S5). These increased frequencies of alterations indicate that eRNA target genes tend to encompass regions with driver events, suggesting their potential as viable therapeutic targets [37].

Considering the significance of enhancers in therapy-resistant cancers and the functional role of eRNAs as units of enhancer activity, we investigated the impacts of eRNAQTL-eRNAs on drug responses in cancers. A total of 194,660 eRNA-drug pairs were identified from the Genomics of Drug Sensitivity in Cancer (GDSC) database [38], and these drugs were further classified according to their pharmaceutical targets (Additional file 1: Table S11). Interestingly, it was found that drugs targeting phosphoinositide 3-kinase/mammalian target of rapamycin (PI3K/mTOR) signaling were broadly associated with multiple eRNAQTL-eRNAs across different cancer types (Fig. 2f; Additional file 2: Fig. S6). These findings are consistent with previous studies demonstrating that the hyperactivation of PI3K/mTOR signaling contributes to cancer metastasis and chemoresistance by regulating the cell cycle and proliferation [39, 40]. Additionally, the clinical relevance of eRNAQTL-eRNAs was evaluated and 250 eRNAs associated with patients’ survival were identified; however, most of them were specific to individual cancer types, indicating a caner-specific pattern (Fig. 2g; Additional file 1: Table S12). Collectively, our findings provide compelling evidence supporting the clinical utility of eRNAs as effective biomarkers and potential therapeutic targets.

### Profiling eRNAQTLs in Chinese CRC patients

**Fig. 3:**
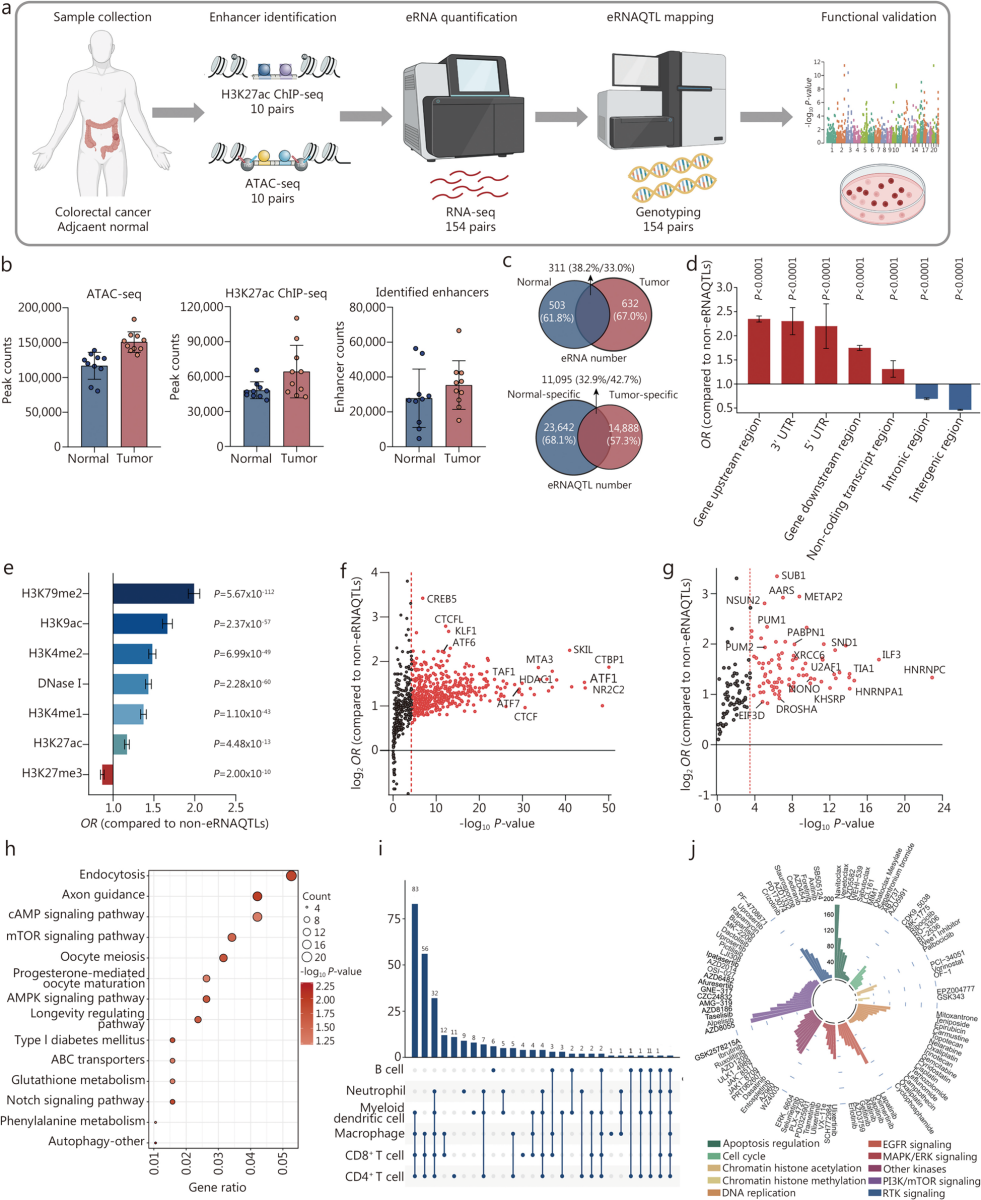
Validation of eRNAQTL profiles in Chinese colorectal cancer (CRC) samples. **a** Flowchart of enhancer identification and eRNAQTLs mapping in our CRC discovery set. ATAC-seq and H3K27ac ChIP-seq signals of 10 pairs of CRC tumor and adjacent normal tissues were integrated to identify active enhancer elements. Then, RNA-seq data of 154 pairs of tumor and normal tissues were mapped to these enhancer regions for quantifying eRNA transcription. By combining genotype data, the first Chinese eRNAQTL profiles were established in CRC. **b** The number of ATAC-seq peaks, H3K27ac ChIP-seq peaks, and active enhancers in 10 pairs of tumor and adjacent normal tissues. **c** Venn diagram showing the number of eRNAs (top) and eRNAQTLs (bottom) identified in 154 pairs of normal and tumor tissues. **d** Enrichment analysis of tumor eRNAQTL SNPs in different genomic categories. *P*-values were calculated by a two-tailed Fisher’s exact test. Bars indicate 95%CI. **e** Enrichment analysis of tumor eRNAQTLs among variants within regulatory elements. *P*-values were calculated by a two-tailed Fisher’s exact test. Bars indicate 95%CI. **f** Plots of -log_10_ *P*-value (X-axis) and -log_2_ *OR* (Y-axis) were obtained from enrichment analysis of tumor eRNAQTLs among variants within TF binding sites. The red dashed line indicates *P* = 0.05/801 (6.24 × 10^−5^) (Bonferroni-corrected *P*-value threshold, binding sites for a total of 801 TFs were tested). **g** Enrichment of eRNAQTL SNPs at RBP binding sites by two-tailed Fisher’s exact test. The red dashed line indicates the Bonferroni-corrected *P*-value threshold according to the number of RBPs tested (150 RBPs). **h** KEGG pathway analysis for target genes of eRNAQTL-eRNAs in CRC. The circle size represents the number of genes enriched in each pathway, and the color indicates the -log_10_ *P*-value of enrichment. **i** Number of eRNAQTL-eRNAs associated with immune cell fractions, calculated by partial correlation coefficient with tumor purity adjusted. **j** Circle plot summarized the number of eRNAQTL-eRNAs associated with pharmaceutical targets in different categories. The count numbers are displayed with different colors by the cancer signaling pathway. ATAC-seq assay for transposase-accessible chromatin with high throughput sequencing, ChIP-seq chromatin immunoprecipitation sequencing, CI confidence interval, RBP RNA binding protein, KEGG Kyoto Encyclopedia of Genes and Genomes, *OR* odds ratio, CRC colorectal cancer, SNP single nucleotide polymorphism, eRNAQTL eRNA quantitative trait locus, MAPK/ERK mitogen-activated protein kinase/extracellular signal-regulated kinase, EGFR epidermal growth factor receptor, RTK receptor tyrosine kinase, PI3K/mTOR phosphoinositide 3-kinase/mammalian target of rapamycin pathway, cAMP cathelicidin antimicrobial peptide, ABC transporters ATP-binding cassette transporters, AMPK adenosine 5’-monophosphate (AMP)-activated protein kinase, mTOR mammalian target of rapamycin

Since eRNAQTLs make a significant contribution to CRC heritability, and the characteristics of eRNAQTLs in Chinese CRC remain largely unexplored, we conducted multi-omics analyses including ATAC-seq, H3K27ac ChIP-seq, RNA-seq, and genotyping with high quality in Chinese population with CRC (Fig. 3a; Additional file 2: Figs. S7, S8). In total, there were an average of 150,889 ATAC-seq peaks and 64,316 H3K27ac ChIP-seq peaks identified in 10 primary tumor tissues, as well as an average of 116,753 ATAC-seq peaks and 48,233 H3K27ac ChIP-seq peaks identified in their corresponding adjacent tissues (Fig. 3b). By computationally integrating the data from ATAC-seq and H3K27ac ChIP-seq for these tissue pairs (primary tumor vs. adjacent), an average of 35,340 enhancers in tumor tissues and 27,798 enhancers in paired normal tissues were identified (Fig. 3b; Additional file 1: Tables S13, S14). To obtain the transcriptional data for these identified enhancers, the RNA-seq reads from 154 CRC tumor/adjacent normal samples were further overlapped with the enhancer regions, and excluded the regions that corresponded to known coding genes. Afterward, eRNAs with relatively high expression levels (RPKM ≥ 0.5 and expressed in more than 50% of samples) were considered detectable eRNAs, resulting in 943 eRNAs being identified in tumor tissues and 814 identified in adjacent normal tissues (Fig. 3c; Additional file 1: Tables S15, S16).

We then established the first genome-wide eRNAQTL map across 154 pairs of CRC and adjacent normal tissues to reveal the important biological function of eRNAQTLs in the Chinese population. By leveraging genotype of germline variants and eRNA expression profiles, a total of 25,983 eRNAQTLs for the tumor-derived tissues and 34,737 eRNAQTLs for the adjacent normal tissues were identified (Fig. 3c; Additional file 1: Tables S17, S18). Consistent with results from the pan-cancer profiles, the eRNAQTLs in our tumor tissues were predominantly localized to enhancer-related functional regions (Fig. 3d, e). There was a significant enrichment of eRNAQTLs in genomic regions bound by modified histones associated with active chromatin marks such as H3K79me2 (*OR* = 1.99, 95%CI 1.87–2.11, *P* = 5.67 × 10^−112^), H3K9ac (*OR* = 1.66, 95%CI 1.56–1.77, *P* = 2.37 × 10^−57^) and H3K4me2 (*OR* = 1.48, 95%CI 1.40–1.56, *P* = 6.99 × 10^−49^). Moreover, a significant enrichment of eRNAQTLs was observed for 455 TFs (801 TFs in total, Fig. 3f) and 84 RBPs (150 RBPs in total, Fig. 3g). Interestingly, the prominent enrichment in TFBS was observed for C-terminal binding protein 1 (CTBP1), nuclear receptor subfamily 2 group C member 2 (NR2C2) and activating transcription factor 1 (ATF1), which are key drivers in CRC (Fig. 3f). To elucidate the molecular functions of eRNAs in cancer development, expression data for eRNAs and mRNAs from 154 CRC tissues were integrated, and co-expression analyses were performed to identify candidate target genes of the eRNAs. Furthermore, pathway enrichment analysis revealed that the genes regulated by eRNAs were implicated in canonical cancer pathways, including endocytosis, axon guidance, and cathelicidin antimicrobial peptide (cAMP), indicating a tendency toward oncogene regulation (Fig. 3h; Additional file 2: Fig. S9). Additionally, it was discovered that 267 eRNAQTL-eRNAs (353 eRNAs in total) were related to substantial immune cell infiltration, with myeloid dendritic cells, macrophages, CD8^+^ T cells and CD4^+^ T cells processing the highest number of eRNAs (Fig. 3i). A total of 7087 eRNA-drug pairs were identified using the GDSC drug dataset, with drugs targeting the PI3K/mTOR signaling pathway ranking highest. Among them, 158 eRNAQTL-eRNAs were linked to AZD8055, an inhibitor targeting the PI3K/mTOR signaling pathway (Fig. 3j). Our assessment of these eRNAs regarding immune cell infiltration and pharmaceutical targets reasserted their potential in clinical utility. These findings were subsequently replicated in CRC patients from TCGA (Additional file 2: Figs. S10, S11).

### Comparison of eRNAQTLs in CRC tissues and adjacent normal tissues from Chinese CRC patients

**Fig. 4:**
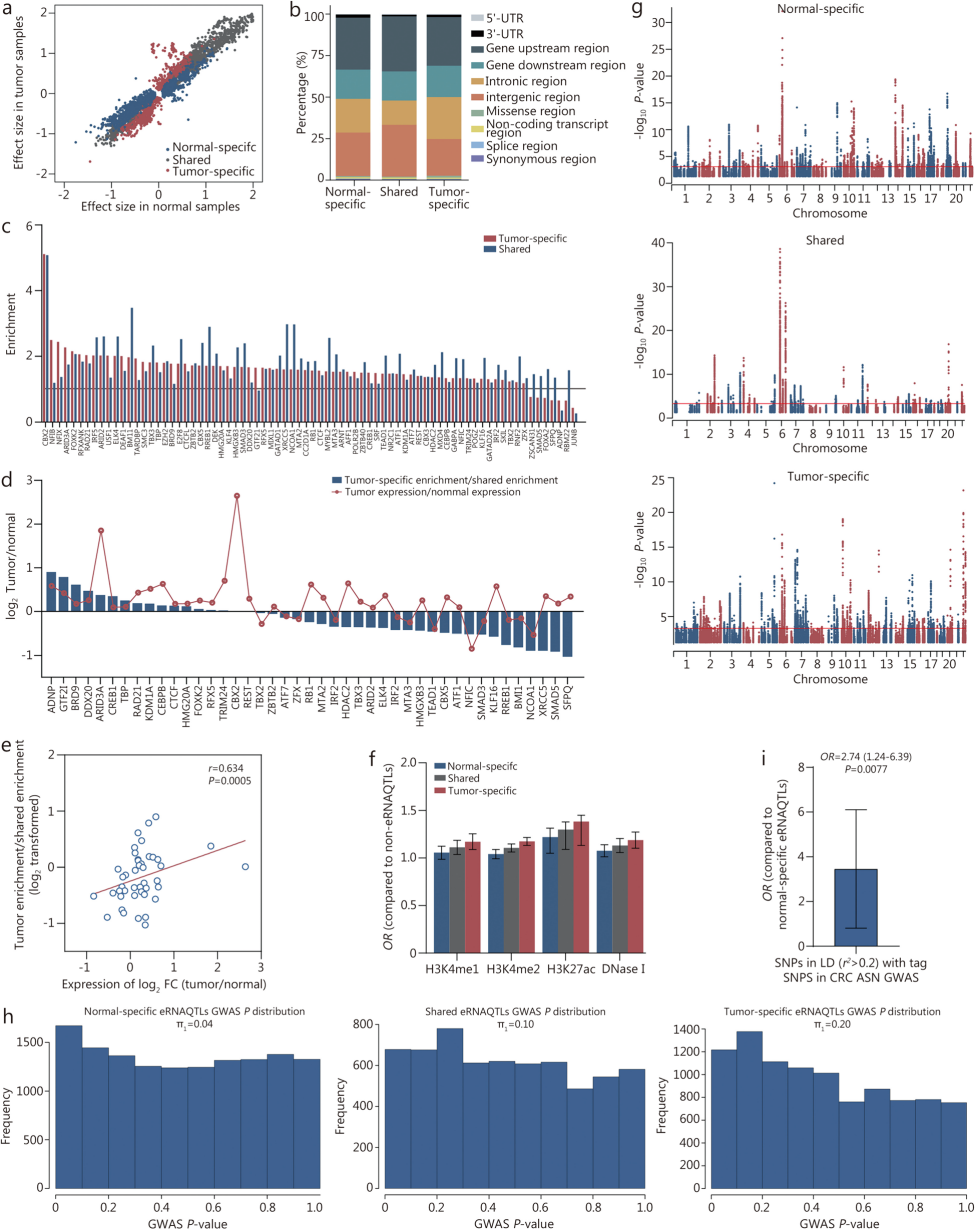
Comparison between eRNAQTLs in normal and tumor-derived colorectal tissues. **a** Effect sizes of eRNAQTLs identified in normal samples vs. tumor samples. **b** The stacked bar charts indicate the percentage of 10 functional categories for each type of eRNAQTLs. **c** The enrichment of tumor-specific eRNAQTLs and shared eRNAQTLs in TF binding sites. The bar plot is ordered by the tumor-specific enrichment. The null (frequency and variant type matched) is represented as the black horizontal line. **d** The ratio of the tumor-specific enrichment to the shared enrichment (in log scale) for significantly differentially expressed transcription factors (TFs) are shown as blue bars. The red line depicts the differential expression fold change of the TFs. **e** Correlation between the enrichment in TF binding sites and fold change (FC) of differentially expressed TFs. **f** Enrichment analysis of eRNAQTLs among variants within 4 epigenomic marks, including H3K4me1, H3K4me2, H3K27ac, and DNase I hypersensitive sites. *P*-values were calculated by a two-tailed Fisher’s exact test. **g** Manhattan plot of -log_10_ *P*-value from the results of three types of eRNAQTLs in our CRC samples (normal-specific eRNAQTLs, shared eRNAQTLs, and tumor-specific eRNAQTLs). The eRNAQTL *P*-values (-log_10_) of the SNPs (Y-axis) are presented according to their chromosomal positions (X-axis). **h** CRC GWAS *P*-value distributions and statistics for normal-specific, shared, and tumor-specific eRNAQTLs. **i** Enrichment analysis of tumor-specific eRNAQTL SNPs among CRC risk loci in Asian populations. The *P*-value was calculated by two-tailed Fisher’s exact tests. *OR* is depicted on the Y-axis and bars indicate 95%CIs. *OR* odds ratio, CRC colorectal cancer, CI confidence interval, ASN Asian, eRNAQTL eRNA quantitative trait locus, SNP single nucleotide polymorphism, GWAS genome-wide association study

Many associations between genetic variants and transcription do not occur at steady state but in specific contexts, which may be partially facilitated by functional elements acting in a state-dependent mode, such as modified histones and TFs [41, 42]. Thus, the identification of tumor-specific variants through comparative QTL analysis in both the tumor and the normal state is an important addition to deciphering the biological mechanisms underlying cancer development. Here, the eRNAQTLs identified in our CRC tissues were compared with adjacent normal tissues. It was found that 57.3% of the tumor eRNAQTLs and 68.1% of normal eRNAQTLs appeared to be tissue-specific, while there were 11,095 shared eRNAQTLs (Fig. 3c; Additional file 1: Table S19). Analyses of the effect sizes of eRNAQTLs showed that the direction of shared eRNAQTL effects remained conserved after tumorigenesis (Fig. 4a). Moreover, similar patterns were observed for the functional distribution of state-specific and shared eRNAQTLs (Fig. 4b). To elucidate whether different regulatory elements drive the differences between tumor-specific and shared eRNAQTLs, the enrichment in regions bound by TFs was examined (Fig. 4c). It was observed that 17 TFs were strongly enriched in the tumor-specific eRNAQTLs, all of which were also significantly upregulated in CRC (Fig. 4d), including canonical cancer-associated TFs such as bromodomain containing 9 (BRD9), cAMP responsive element binding protein 1 (CREB1) and CCAAT enhancer binding protein beta (CEBPB). Correspondingly, there was a significant positive correlation between the ratio of tumor-specific eRNAQTL enrichment to shared eRNAQTL enrichment and fold change (FC) in the expression of the corresponding TFs (*r* = 0.634, *P* = 0.0005; Fig. 4e), indicating that regulatory switches might explain some of the tumor-specific eRNAQTL effects. The tumor-specific eRNAQTLs had stronger enrichment in active histone modification (Fig. 4f). Furthermore, the Manhattan plots revealed distinct distribution patterns for three types of eRNAQTLs, with prominent loci located on different chromosomes, prompting further investigation into their various functions in CRC (Fig. 4g). The highest levels of enrichment for low GWAS *P*-values were found in tumor-specific eRNAQTLs (π_1_ of 0.20 vs. 0.10 in shared eRNAQTLs, 0.04 in normal-specific eRNAQTLs; Fig. 4h), indicating that a proportion of the CRC GWAS signals were cis-regulatory variants exclusively active in tumors. Additionally, it was discovered that CRC GWAS signals from CRC patients with Asian ancestry were enriched in tumor-specific eRNAQTLs compared to normal-specific ones (Fig. 4i), suggesting that tumor-specific eRNAQTLs contribute significantly more to cancer heritability. Overall, these results reveal that certain activated functional roles played by specific tumor-state-associated eRNAQTLs might contribute to CRC tumorigenesis.

### The eRNAQTL rs3094296 contributes to a decreased risk of CRC in multiple populations

**Fig. 5:**
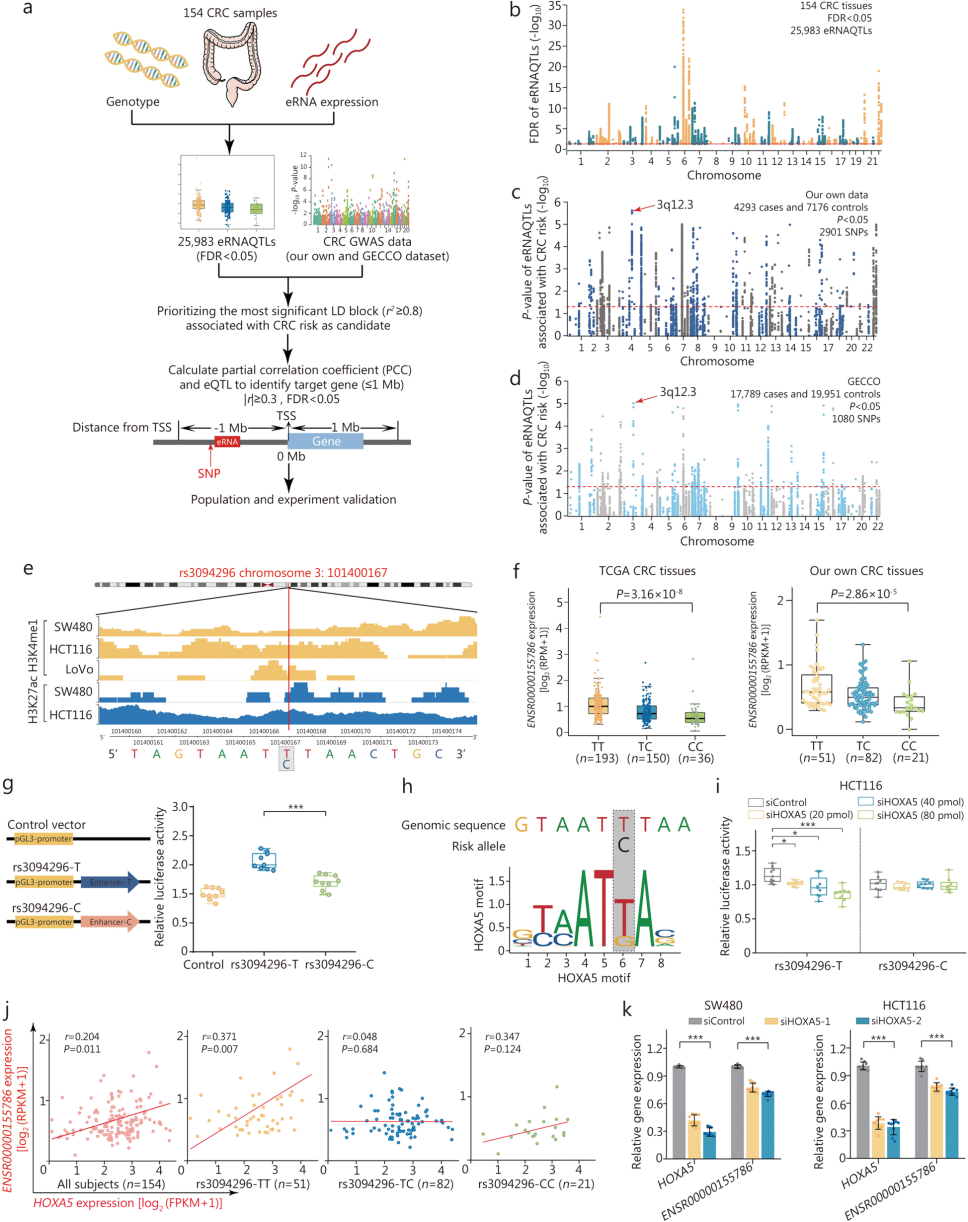
eRNAQTL rs3094296 promotes eRNA *ENSR00000155786* expression mediated by TF HOXA5. **a** Workflow to identify the candidate variant by integrating eRNAQTL analysis and CRC GWAS data, the target gene was selected by combining co-expression analysis and eQTL result in CRC. **b** Manhattan plot of eRNAQTL results in 154 CRC samples. The red line shows the FDR < 0.05 threshold. **c** Manhattan plot for associations between eRNAQTLs and CRC risk from the Chinese population consisting of 4293 cases and 7176 controls. **d** Validation in European populations from GECCO, with a combined sample size of 17,789 cases and 19,951 controls. *P*-values were calculated by an unconditional logistical regression analysis with an additive model adjusting for gender, age, smoking, and drinking status. *P* < 0.05 was considered statistically significant (indicated by the red line). **e** Epigenetic tracks obtained from the Cistrome database show the enrichment of enhancer markers (H3K4me1 and H3K27ac peaks) in the rs3094296 region. **f** eRNAQTL analysis demonstrates the correlation between the rs3094296 genotype and the expression of *ENSR00000155786* in the TCGA CRC samples and our own CRC tissues. Data were shown as the median (minimum to maximum). **g** Relative reporter gene activity of the vectors containing the rs3094296-T or rs3094296-C allele in HCT116 cell. **h** The rs3094296-T allele resides within the HOXA5 binding motif. **i** The effect of *HOXA5* knockdown on the relative luciferase activity of vectors containing the rs3094296-T or rs3094296-C allele in HCT116 cell. Data were presented as the median (minimum to maximum) from three repeated experiments, each has three technical replicates. *P*-values were calculated by a two-sided Student’s *t*-test. **j** Scatter plots show the correlations between *HOXA5* and *ENSR00000155786* expression in our CRC tumor tissues and stratified by SNP rs3094296 genotype. All *P*-values and correlation coefficients were calculated by Spearman’s correlation analysis. **k** Histogram displays the effect of *HOXA5* knockdown on the expression of *ENSR00000155786* in SW480 and HCT116 cells. Data were presented as the mean ± SD from three repeated experiments with three technical replicates. All *P*-values were calculated using a two-sided Student’s *t*-test. ^*^P < 0.05, ^***^P < 0.001. CRC colorectal cancer, FDR false discovery rate, GECCO Genetics and Epidemiology of Colorectal Cancer Consortium, LD linkage disequilibrium, FPKM fragments per kilobase of exon model per million mapped fragments, RPKM reads per kilobase per million mapped reads, HOXA5 homeobox A5, SD standard deviation

To validate the role of eRNA as a crucial link between genetics and cancer risk, we conducted a comprehensive analysis involving eRNAQTLs, a large-scale population study and a series of functional experiments in CRC tissues (Fig. 5a). A total of 25,983 eRNAQTLs identified in our CRC tissues were included in the population study (Fig. 5b). In the discovery stage, a large-scale genome-wide analysis of 4293 cases and 7176 controls from China was performed (Additional file 2: Fig. S1). Interestingly, there were 11 SNPs in strong LD (*r*^2^ ≥ 0.8) located in the 3q12.3 region (Additional file 2: Fig. S12), which was the most significant loci associated with CRC risk (*P* < 1 × 10^−4^, Fig. 5c). This locus was also discovered in a European population study encompassing 17,789 cases and 19,951 controls from GECCO cohort [23] (Fig. 5d). To prioritize the variant with potentially greater regulatory function, a functional annotation was performed for eRNAQTLs in this LD block using bioinformatic tools, including Haploreg, RegulomeDB and CistromeDB. It was found that the eRNAQTL variant rs3094296 was enriched within classic enhancer features and active histone modification peaks (Fig. 5e). Hence, rs3094296 was selected as a candidate for subsequent validation.

Replication stage I was conducted in independent Chinese and European populations, consisting of 1524 cases and 1522 controls from Beijing, and 1233 cases and 6165 controls from PLCO [24], respectively. It was illustrated that the T allele of the eRNAQTL rs3094296 conferred a decreased risk of CRC. These associations were further validated in replication stage II with 4500 cases and 8500 controls from Wuhan, and 5246 cases and 26,230 controls from UKB (Additional file 2: Fig. S1). Finally, through the combined analysis of data from the discovery and replication stages, convincing evidence was presented for the link between rs3094296 and CRC risk in both Chinese (*OR* = 0.91, 95%CI 0.88–0.95, *P* = 2.92 × 10^−7^ in additive model) and European populations (*OR* = 0.92, 95%CI 0.88–0.95, *P* = 4.61 × 10^−6^ in additive model, Table 1).

**Table 1:** Association analyses between individual SNP and colorectal cancer risk in the three phases and combined samples

Stages		Asian/Chinese (*n* = 27,515)			European population (*n* = 76,614)		
		Cases/Control	*OR* (95%CI)	*P*	Cases/Control	*OR* (95%CI)	*P*
Discovery stage (*n* = 49,209)							
CC		458/603^a^	1		996/998^b^	1	
Recessive (TT)		1960/3494^a^	0.89 (0.82–0.96)	1.90 × 10^−3^	11,633/13,430^b^	0.93 (0.89–0.97)	5.81 × 10^−4^
Additive model			0.88 (0.83–0.94)	2.69 × 10^−5^		0.94 (0.90–0.97)	2.45 × 10^−4^
Replication stage I (*n* = 10,444)							
CC		274/228^c^	1		124/570^d^	1	
Recessive (TT)		614/705^c^	0.74 (0.64–0.86)	1.02 × 10^−4^	577/3253^d^	0.78 (0.69–0.88)	1.33 × 10^−4^
Additive model			0.84 (0.75–0.92)	3.74 × 10^−4^		0.85 (0.78–0.94)	8.45 × 10^−4^
Replication stage II (*n* = 44,476)							
CC		872/1633^e^	1		677/3065^f^	1	
Recessive (TT)		1800/3611^e^	0.89 (0.83–0.96)	3.81 × 10^−3^	2152/11,317^f^	0.92 (0.86–0.97)	4.44 × 10^−3^
Additive model			0.94 (0.90–0.99)	1.76 × 10^−2^		0.93 (0.89–0.97)	1.01 × 10^−3^
Combined study (*n* = 104,129)							
CC		1604/2464	1		1797/4633	1	
Recessive (TT)		4374/7810	0.90 (0.86–0.95)	6.49 × 10^−5^	14,362/28,000	0.89 (0.84–0.94)	9.76 × 10^−6^
Additive model			0.91 (0.88–0.95)	2.92 × 10^−7^		0.92 (0.88–0.95)	4.61 × 10^−6^

^a^Chinese individuals collected from the Chinese Academy of Medical Sciences, Tongji Hospital of the Huazhong University of Science, and Technology, Zhongnan Hospital of Wuhan University, and Renmin Hospital of Wuhan University

^b^European individuals in GECCO

^c^Chinese individuals collected from Chinese Academy of Medical Sciences

^d^European individuals from PLCO

^e^Chinese individuals collected from Tongji Hospital of Huazhong University of Science and Technology

^f^European individuals from UKB. *OR* and 95%CI calculations were conducted under the assumption that variant alleles were risk alleles

### eRNAQTL rs3094296 promotes eRNA *ENSR00000155786* expression mediated by HOXA5

Next, we investigated the potential molecular mechanisms underlying the contribution of eRNAQTL rs3094296 to CRC risk. It was found that eRNAQTL rs3094296 was robustly correlated with the expression of eRNA *ENSR00000155786* in tumor tissues, while no significant correlation was found in normal tissues (Fig. 5f). Moreover, cell lines carrying the rs3094296-TT genotype (SW480 and HCT116) demonstrated higher expression levels of *ENSR00000155786* compared to HCT15 cells carrying the rs3094296-TC genotype (Additional file 2: Fig. S13a). Subsequently, a luciferase vector containing different alleles was constructed to assess the enhancer activity. The results indicated that the rs3094296-T allele group exhibited higher luciferase activity than the rs3094296-C allele in HCT116 and SW480 cell lines, while this difference was not statistically significant in HIEC-6 cells (Fig. 5g; Additional file 2: Fig. S13b), suggesting an allele-specific effect eRNAQTL rs3094296 on regulating eRNA *ENSR00000155786* expression in CRC.

Growing evidence indicates that the transcription of eRNAs is primarily regulated by the binding of TF to enhancer elements [6, 31]. Simultaneously, the above analyses also revealed a significant enrichment of TFBSs in eRNAQTLs, suggesting that TF binding affinity may influence eRNA expression. To verify our hypothesis, we conducted TF motif analysis using JASPAR (https://jaspar.elixir.no/), and identified that the HOXA5 motif specifically binds to the rs3094296-T allele (Fig. 5h). Furthermore, the knockdown of *HOXA5* remarkably attenuated enhancer activity of rs3094296-T allele in a dose-dependent manner in HCT116 and SW480 cell lines, but had no effect on HIEC-6 cells (Fig. 5i; Additional file 2: Fig. S14), indicating preferential binding of HOXA5 to rs3094296-T allele in the tumor states. Moreover, it was observed that *HOXA5* was positively correlated with *ENSR00000155786* expression in an allele-specific manner in both our CRC samples (Fig. 5j) and TCGA CRC samples (Additional file 2: Fig. S15). Additionally, knockdown of *HOXA5* in SW480 and HCT116 cell lines resulted in decreased expression of *ENSR00000155786*, whereas there was no significant difference in HIEC-6 cells (Fig. 5k; Additional file 2: Fig. S16). These results collectively suggest that the allele-specific effect of eRNAQTL rs3094296 on eRNA *ENSR00000155786* expression is mediated by HOXA5.

### eRNA *ENSR00000155786* inhibits cell proliferation by activating *SENP7* expression

**Fig. 6:**
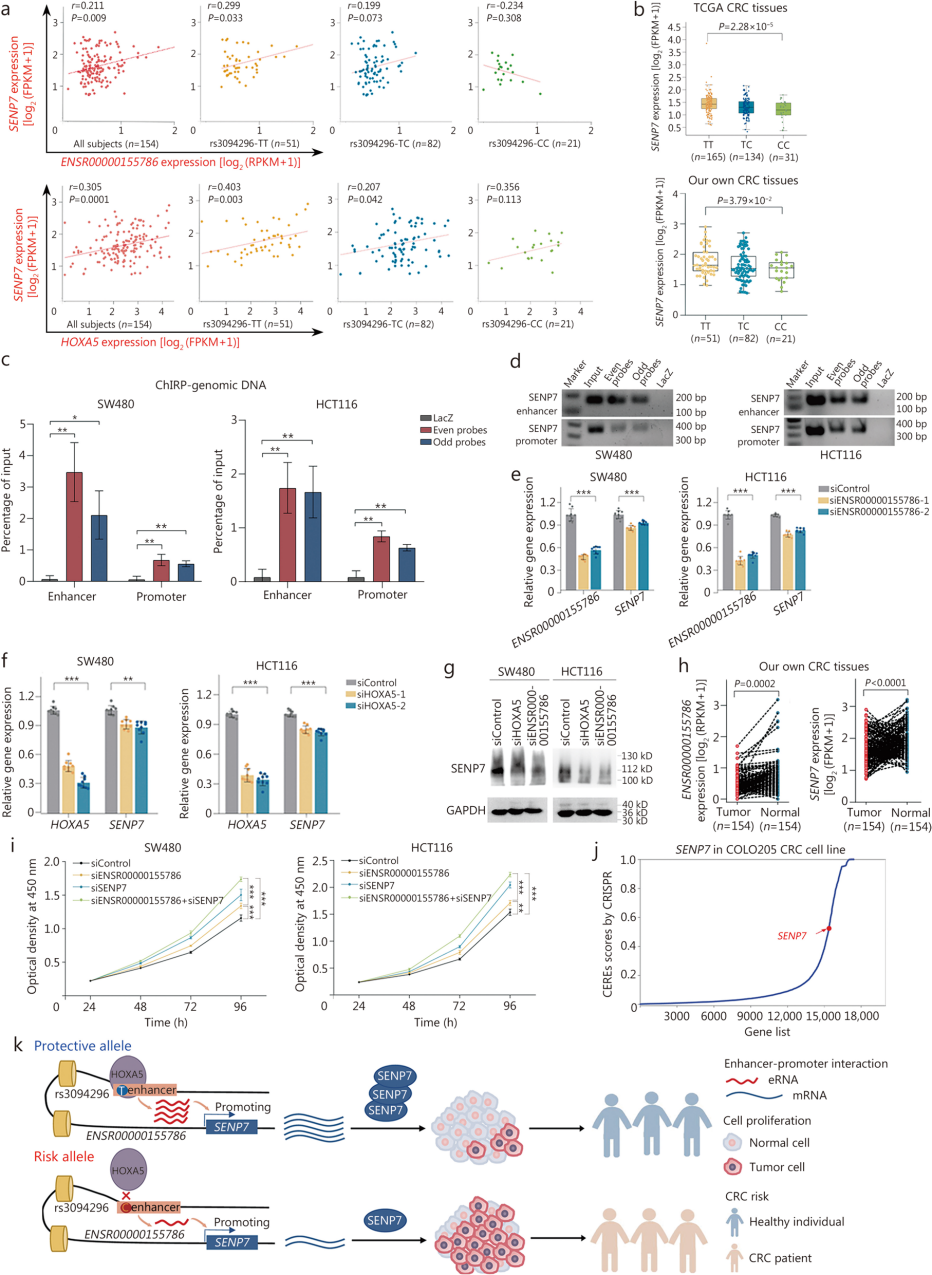
eRNA *ENSR00000155786* inhibits colorectal cancer (CRC) cell proliferation by activating *SENP7* expression. **a** Scatter plots show the correlations between *ENSR00000155786* and *SENP7* expression, as well as the correlations between *HOXA5* and *SENP7* expression in our CRC tumor tissues both of them, are stratified by SNP rs3094296 genotype. All *P*-values and correlation coefficients were calculated by Spearman’s correlation analysis. **b** eQTL analyses demonstrate the correlation between the rs3094296 genotype and the expression of *SENP7* in the TCGA CRC samples and our own CRC tissues. Data were shown as the median (minimum to maximum). ChIRP assay was performed using two different sets of antisense probes (“even group” and “odd group”) that detected *ENSR00000155786* or control probes (anti-LacZ) in CRC cell lines. The enrichment of genomic DNA in the ChIRP and input samples was measured by qPCR (**c**) and agarose gel electrophoresis (**d**). Data were shown as the mean ± SD. *P*-values were calculated by a two-sided Student’s *t-*test. The histogram displays the effect of *ENSR00000155786* knockdown on the expression of *SENP7* (**e**) and the effect of *HOXA5* knockdown on the expression of *SENP7* (**f**) in SW480 and HCT116 cells. Data were presented as the mean ± SD from three repeated experiments with three technical replicates. All *P*-values were calculated using a two-sided Student’s *t*-test. **g** Western blotting analysis shows that the knockdown of *HOXA5* and *ENSR00000155786* reduced the protein expression level of *SENP7* in SW480 and HCT116 cells. **h** *ENSR00000155786* and *SENP7* are significantly decreased in tumor tissues compared with normal tissues from our own CRC tissues. *P*-values were calculated by a paired two-sided Student’s *t*-test. **i** Cell proliferation assay with knockdown of *ENSR00000155786* and *SENP7* in SW480 and HCT116 cells. Results were shown as the means ± SEM from three experiments, each with six replicates. *P*-values were calculated from a two-sided Student’s *t*-test by comparing with controls in 96 h. **j ***SENP7* is essential for cell growth with higher CERE scores in COLO205 CRC cell lines from the data of genome-wide CRISPR/Cas9-based loss-of-function screen. Higher CERE scores demonstrate an elevated dependency of cell viability on given genes. **k** The graph shows the mechanism that eRNAQTL rs3094296 contributes to a decreased risk of CRC by playing allele-specific effect to transcribe eRNA *ENSR00000155786*, which further exerts a transcriptional activator promoting target gene *SENP7* expression, then these two genes display a synergistic effect to inhibit tumor cells proliferation. ^*^P < 0.05, ^**^P < 0.01, ^***^P < 0.001. FPKM fragments per kilobase of exon model per million mapped fragments, RPKM reads per kilobase per million mapped reads, HOXA5 homeobox A5, SENP7 sentrin-specific protease 7, ChIRP chromatin isolation by RNA purification, CERES CRISPR enrichment-based screening, SEM standard error of the mean

To elucidate the molecular functions of eRNA in cancer development, we performed a co-expression analysis to dissect downstream target genes regulated by eRNA *ENSR00000155786*. The expression levels of *ENSR00000155786* and *HOXA5* were significantly associated with *SENP7* expression in our dataset (Fig. 6a) as well as TCGA CRC samples (Additional file 2: Fig. S17). Notably, there was also a positive correlation between the eRNAQTL rs3094296 and the expression of *SENP7* in both cohorts (Fig. 6b). Consistent with this result, the cell lines (HCT116 and SW480) carrying rs3094296-TT genotype exhibited higher *SENP7* expression, compared to the HCT15 cell line carrying rs3094296-TC genotype (Additional file 2: Fig. S18). Furthermore, ChIRP analysis revealed significant enrichment of *ENSR00000155786* at active enhancer and promoter regions of *SENP7* in both probe groups compared to the LacZ probe group (Fig. 6c, d). To further understand the functional role of eRNA and validate its regulation on *SENP7* transcription, knockdown experiments targeting *ENSR00000155786* showed a significant decrease in both mRNA and protein levels of *SENP7* (Fig. 6e; Additional file 2: Fig. S19a). Similarly, the knockdown of *HOXA5* also resulted in a significant reduction in *SENP7* expression in CRC cell lines, but had no effect on HIEC-6 cells (Fig. 6f, g; Additional file 2: Fig. S19b). Altogether, these results provide evidence that eRNA *ENSR00000155786* acts as a transcriptional activator regulating the expression of neighboring gene *SENP7*.

Subsequently, we explored the functional roles of *ENSR00000155786* and *SENP7* in CRC pathogenesis. In both our and TCGA CRC patients, tumor tissues exhibited significant downregulation of *ENSR00000155786* and *SENP7* compared to normal tissues (Fig. 6h; Additional file 2: Fig. S20). Furthermore, the knockdown of either *ENSR00000155786* or *SENP7* remarkably enhanced the proliferation rates of SW480, HCT116, and HIEC-6 cells (Fig. 6i; Additional file 2: Fig. S21). Interestingly, these two genes displayed a synergistic effect on cell proliferation. Consistent with these findings, genome-wide CRISPR/Cas9-based loss-of-function screening data of the COLO205 CRC cell line confirmed the essential role of *SENP7* in cell proliferation (Fig. 6j) [43]. Collectively, these results indicate that *ENSR00000155786* and *SENP7* function as potential tumor suppressor genes in CRC tumorigenesis and hold promise as therapeutic targets (Fig. 6k).

### A user-friendly database for integrative analysis of eRNAQTLs in cancer

**Fig. 7:**
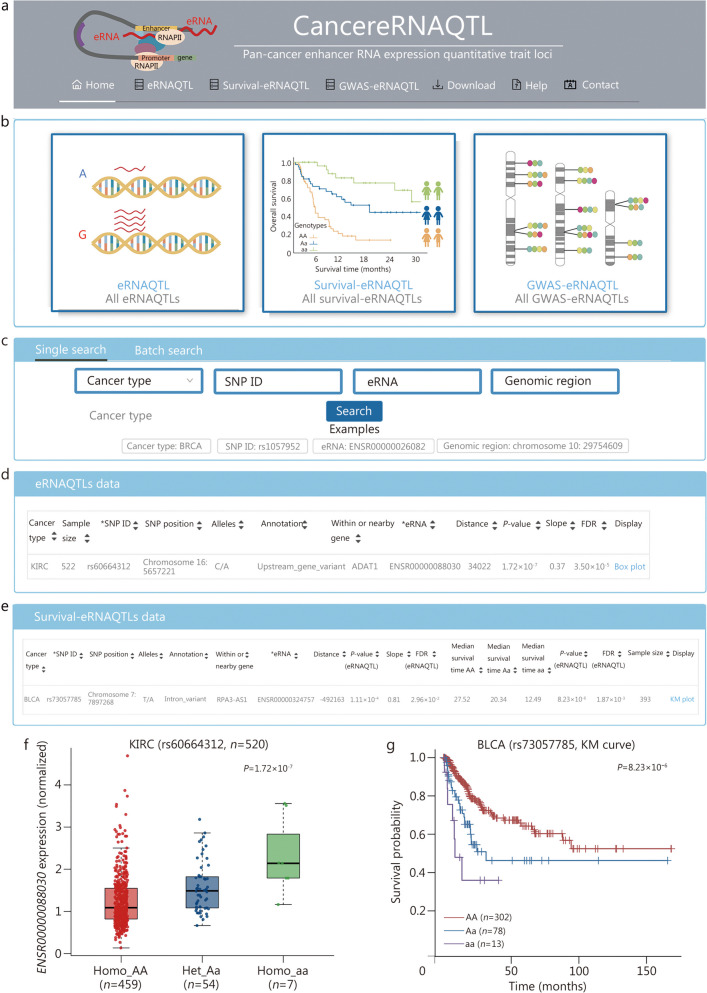
Overview and functional modules of CancereRNAQTL database. **a** Browser bar in CancereRNAQTL. **b** Three modules in CancereRNAQTL, including eRNAQTLs, survival-eRNAQTLs, and GWAS-eRNAQTLs. **c** The single and batch search boxes in CancereRNAQTL. **d** The eRNAQTL search module in CancereRNAQTL. **e** The survival-eRNAQTL search module in CancereRNAQTL. **f** An example of eRNAQTL results on the “eRNAQTL” page and the corresponding boxplot. **g** An example of survival-eRNAQTL results in the “survival-eRNAQTL” page and the corresponding KM plot. BLCA bladder urothelial carcinoma, KIRC kidney renal clear cell carcinoma, KM Kaplan-Meier, eRNAQTL eRNA quantitative trait locus, SNP single nucleotide polymorphism, GWAS genome-wide association study

To facilitate extensive research in the exploration of eRNAQTL function, we have developed a user-friendly database called CancereRNAQTL (http://canernaqtl.whu.edu.cn/#/). This comprehensive resource consists of three interactive modules: eRNAQTLs, survival-eRNAQTLs, and GWAS-eRNAQTLs (Fig. 7a, b). Users can perform both “single search” and “batch search” for comprehensive queries (Fig. 7c). In the eRNAQTL module, users can explore eRNAQTL results based on cancer types, SNP ID, and specific eRNA (Fig. 7d). The survival-eRNAQTL module displayed eRNAQTLs associated with patient survival (Fig. 7e). We embedded boxplots to illustrate the association between eRNAQTL genotypes and eRNA expression for each record (Fig. 7f). Additionally, KM plots are utilized to depict survival correlation (Fig. 7g). Furthermore, extension pages provide a list of eRNAQTLs that can impact mRNA expression while merging with existing eQTL results. The CancereRNAQLT database also incorporates target genes, clinical relevance, and drug targets of identified eRNAs, which are analyzed from multi-omics data, such as transcriptome information, clinical data sets, and pharmacogenomics data. In summary, CancereRNAQTL is an all-encompassing resource for researchers in genetic and cancer studies.

## Discussion

In complex genetic traits such as cancers, most genetic risk variants are located in non-coding regions, indicating that transcriptional regulatory mechanisms may mediate the effects of these genetic variations on traits [44]. Given that enhancers outnumber genes and multiple enhancers can regulate the same gene, eQTL analysis might overlook crucial information regarding the genetic mechanisms mediated by enhancers [45, 46]. Therefore, gaining a deeper understanding of eRNA expression regulation could enhance our understanding of complex genetic traits. In this study, we leveraged eRNA expression profiles and genotypes from 8757 samples obtained from TCGA to identify 300,112 eRNAQTLs across 30 cancer types. Our integration with multi-omics data comprehensively confirmed that eRNAQTLs were enriched in transcriptional features, and explained a significant portion of cancer heritability. Notably, we expanded this eRNAQTL profile to Chinese CRC patients and identified 25,983 eRNAQTLs in tumor tissues and 34,737 eRNAQTLs in adjacent normal tissues. Furthermore, through large-scale cross-ancestry studies involving 34,585 CRC patients and 69,544 controls, an eRNAQTL rs3094296-T was found to significantly decrease the risk of CRC. Mechanistically speaking, rs3094296 modulated the binding of TF HOXA5 in an allele-dependent manner to promote the expression of eRNA *ENSR00000155786* and mRNA *SENP7*, which synergistically suppressed tumor cell proliferation. Moreover, a user-friendly database called CancereRNAQTL was established to enable future study in this field. Overall, the comprehensive analyses presented here provide new evidence supporting the notion that eRNA transcription may bridge the gap between genetic variation and complex phenotypes.

QTL analysis has linked genetic variants to eRNA expression, prompting our research into the potential regulatory mechanisms by which these SNPs exert their functions. Our observation that eRNAQTLs were substantially enriched in enhancer-related genomic features and epigenetic markers, such as DNase I hypersensitivity site, H3K4me1, and H3K27ac markers [47], support the connection between chromatin status and transcriptional activation of eRNAs. Moreover, the significant enrichment of eRNAQTLs in TFBSs indicated that TFs played important roles in regulating enhancer transcription. Importantly, we have demonstrated that TF HOXA5 can regulate eRNA expression in an allele-specific manner in CRC. These findings suggest that variants within enhancer regions may lead to either loss or gain of enhancer activity by modulating chromatin accessibility, histone modifications, and the binding affinity of TFs, thereby influencing the transcriptional regulation of eRNAs.

Emerging studies have found that associations between genotypes and eRNA transcription offered a potential mechanism for gene regulation, providing important insights into disease susceptibility [17, 19]. Similarly, our integration analysis of eRNAQTLs and GWAS showed that eRNAs play a critical role in bridging genetics and human cancers. Given that eRNA levels can generally maintain cell type-specific signals, whereas mRNA expression levels from bulk RNA-seq data reflect the average signal across numerous cell types within a tumor, the defined eRNA loci map presented here can serve as an essential supplement for studying disease heritability. Our subsequent investigation on heritability demonstrated that eRNAQTLs can explain a substantial proportion of cancer heritability independently from eQTLs. Moreover, the eRNAQTL mapping in our matched tumor and normal CRC samples demonstrated that eRNAQTLs that were activated in the tumor state were more likely to contribute to CRC tumorigenesis. Collectively, our findings indicate that eRNAQTLs capture different aspects of cancer genetics, and add to growing evidence that a proportion of GWAS risk variants contribute to disease risk through their involvement in regulating eRNAs.

The identification of enhancer-gene regulatory networks holds significant importance in both mechanistic and translational research [48]. Most eRNAQTL-eRNAs we identified functioned as regulators of clinically actionable genes and immune checkpoints, particularly in canonical cancer-immune pathways. This provides additional evidence for the functional role of eRNAs in oncogene dysregulation during cancer development. Moreover, eRNAQTL-eRNAs exhibited a strong expression pattern specific to each cancer type and had active connections with immune cell infiltration and response to anticancer drugs, pinpointing that they have potential as biomarkers or therapeutic targets in cancers. More specifically, we demonstrated that *ENSR00000155786*, an eRNA regulated by eRNAQTL rs3094296, functioned as a transcriptional activator that promotes the expression of the target gene *SENP7*. These two genes synergistically suppressed the malignant phenotypes of CRC. *SENP7* (SUMO-specific peptidase 7) encodes a protease involved in deconjugating SUMOs from their substrates and regulating DNA repair and innate immune responses [49]. Recent findings also revealed that *SENP7* plays an antitumor role by sensing oxidative stress to maintain the metabolic fitness and effector function of CD8^+^ T cells [50]. Similarly, *ENSR00000155786* and *SENP7* were found to be downregulated in both the TCGA dataset and our CRC tissues, exhibiting a synergistic effect on tumor cell proliferation. These findings uncover that enhancer overactivation can contribute to oncogenesis and suggest the potential of targeting eRNAs as a novel anticancer therapeutic strategy.

Several challenges need to be addressed before eRNAQTLs can be effectively utilized for elucidating the genetic basis of diseases. Firstly, conventional RNA-seq techniques only have the capability to detect a subset of polyadenylated eRNAs at their steady state due to limitations in polyA selection and relatively low sequencing depth. Moreover, there is still a lack of understanding regarding non-polyadenylated eRNAs. Therefore, it is imperative to explore alternative technologies such as global run-on sequencing (GRO-seq) and precision run-on sequencing (PRO-seq) to achieve a more comprehensive view of eRNAs in the future. Secondly, although our study has identified a set of tumor-specific eRNAQTLs, the underlying mechanisms driving these specific eRNAQTL effects remain unexplored. Considering that 3D chromatin topology and accessibility play crucial roles in regulating gene expression during development and disease progression, it becomes essential to investigate genomic topology and local chromatin accessibility at different stages to illustrate how variations in regulatory elements impact gene expression plasticity. Additionally, it is necessary to focus on mapping eRNAQTLs across various cell types by utilizing single-cell technology.

## Conclusion

We conducted a systematic analysis of the genetic effect of eRNA expression across 30 different cancer types, resulting in the creation of a comprehensive eRNAQTL resource that can facilitate the interpretation of plausible variants associated with cancers. Our in-depth depiction highlights the importance of eRNAQTLs and their target eRNAs in tumorigenesis and clinical application. Importantly, we quantified the expression of enhancer regions in CRC tissues from Chinese patients and established the first Chinese eRNAQTL profiles. Furthermore, we identified an association between the eRNAQTL rs3094296 and risk of CRC through large-scale, multi-ethnic population studies comprising 34,585 cases and 69,544 controls. Mechanistically, this variant exhibits an allele-specific effect in promoting transcription of both the eRNA *ENSR00000155786* and mRNA *SENP7*, which synergistically suppress tumor cell proliferation. Our study uncovers that genetic variation influencing eRNAs expression further enhances our understanding of transcriptional regulatory mechanisms, and provides additional insights into disease etiology.

### Supplementary Information

** Additional file 1: Table S1** List of eRNAs in 30 cancer types from TCGA. **Table S2** Summary of characteristics of individuals in the replication stage. **Table S3** Probes or primers sequence used in the study. **Table S4 **Summary of eRNAQTLs in 30 cancers. **Table S5** Enrichment of eRNAQTLs in the histone modification peaks. **Table S6** Enrichment of eRNAQTLs in the binding site of transcription factors. **Table S7** Enrichment of eRNAQTLs in the binding site of RNA binding proteins. **Table S8** Expression patterns of eRNAs in 30 cancers from TCGA. **Table S9** Putative target genes of eRNAs in 30 cancers from TCGA. **Table S10** Correlations between eRNAs and immune infiltration in 30 cancers from TCGA. **Table S11** Correlations between eRNAs and drug response in 30 cancers from TCGA. **Table S12** Correlations between eRNAs and patients’ survival in 30 cancers from TCGA. **Table S13** Enhancers identified in our colorectal cancer tissues. **Table S14** Enhancers identified in our adjacent normal tissues. **Table S15** Enhancer RNAs identified in our colorectal cancer tissues. **Table S16** Enhancer RNAs identified in our adjacent normal tissues. **Table S17** eRNAQTLs identified in our colorectal cancer tissues. **Table S18** eRNAQTLs identified in our adjacent normal tissues. **Table S19** Tissue specificity of eRNAQTLs identified in our tissues.

** Additional file 2: Materials and methods.** **Fig. S1 **The three-stage studies design and workflow. **Fig. S2** Overview of methods and quality control pipeline for eRNAQTL identification, integrative annotation, and validation. **Fig. S3** Characterization of eRNAQTLs in TCGA 30 cancer types. **Fig. S4** Pathway enrichment analyses for putative target genes of eRNAs. **Fig. S5** The frequencies of amplification and deletion in eRNAQTL-eRNAs’ putative target genes. **Fig. S6** Enrichment analysis on the proportion of eRNAQTL-eRNAs associated with drug response. **Fig. S7** Quality assessment of the high-throughput sequence data in 10 matched colorectal cancer and normal samples. **Fig. S8** Quality assessment of the RNA-sequencing data in 154 matched colorectal cancer and normal samples. **Fig. S9** GO analysis for target genes of eRNAQTL-eRNAs in our CRC tissues. **Fig. S10** Genomic and functional characterization of identified eRNAQTLs in colorectal cancer from TCGA dataset. **Fig. S11** Characterization of eRNAQTL-eRNAs and putative target genes in colorectal cancer from TCGA dataset. **Fig. S12** LD block plot (*r*^2^ ≥ 0.8) showing the *r*^2^ value of candidate variants. **Fig. S13** Relative expression of *ENSR00000155786* in three cell lines. **Fig. S14** Effect of HOXA5 knockdown on the relative luciferase activity of vectors containing the rs3094296-T or rs3094296-C allele in SW480 and HIEC-6 cell lines. **Fig. S15** The correlations between *ENSR00000155786* and *HOXA5* expression in all TCGA CRC samples stratified by SNP rs3094296 genotype. **Fig. S16** The effect of *HOXA5* knockdown on the expression level of *ENSR00000155786* in HIEC-6 cells. **Fig. S17** Correlations of *SENP7* expression with *ENSR00000155786* and *HOXA5* expression in TCGA CRC samples. **Fig. S18** Relative expression of *SENP7* in HCT116 (rs3094296-TT) and SW480 (rs3094296-TT) cell lines compared to HCT15 (rs3094296-TC) cell line. **Fig. S19** The expression level of *SENP7* and *ENSR00000155786* in HIEC-6 cells. **Fig. S20** Differential expression of *ENSR00000155786* and *SENP7* in TCGA CRC samples. **Fig. S21** Cell proliferation assay with knockdown of *ENSR00000155786* and *SENP7* in HIEC-6 cells.

## Data Availability

Enhancer RNA expression profile of TCGA samples is available in the eRic database (https://hanlab.uth.edu/eRic). GWAS summary results were obtained from the NHGRI GWAS catalog (http://www.ebi.ac.uk/gwas/). ChIP-seq peaks, TF-binding sites, and eCLIP-seq data among human cancer cell lines were downloaded from the ENCODE portal (https://www.encodeproject.org/data/annotations/). Expression profile data and drug sensitivity data of cancer cell lines were obtained from the GDSC (https://www.cancerrxgene.org/). Genotype data of GECCO was downloaded from dbGaP under accession numbers phs001078.v1.p1, phs001315.v1.p1, and phs001415.v1.p1. Genotype data of PLCO was downloaded from dbGaP under accession numbers phs000346.v2.p2, phs001554.v1.p1, phs001286.v2.p2, and phs001524.v1.p1. Genotype data from UK Biobank (http://www.ukbiobank.ac.uk/) was obtained under application No. 94939. Data supporting the findings of the study are uploaded to our online data portal or presented in the Additional file 2: Materials and methods. All software and R packages used in the study are publicly available as described in the methods and Additional file 2: Materials and methods.

## Abbreviations

ATAC-seq: Assay for transposase-accessible chromatin with high throughput sequencing
BLCA: Bladder urothelial carcinoma
ChIP-seq: Chromatin immunoprecipitation sequencing
CAGE-seq: Cap analysis of gene expression sequencing
CRC: Colorectal cancer
ChIRP: Chromatin isolation by RNA purification
CHOL: Cholangiocarcinoma
eRNA: Enhancer RNA
eRNAQTL: eRNA quantitative trait locus
ENCODE: Encyclopedia of DNA elements
FANTOM: Functional annotation of the mammalian genome
FDR: False discovery rate
GECCO: Genetics and epidemiology of colorectal cancer consortium
GWAS: Genome-wide association study
GAPDH: Glyceraldehyde-3-phosphate dehydrogenase
GSEA: Gene set enrichment analysis
GO: Gene ontology
GDSC: Genomics of drug sensitivity in cancer
KEGG: Kyoto encyclopedia of genes and genomes
LD: Linkage disequilibrium
MAF: Minor allele frequency
OR: Odds ratio
PI3K/mTOR: Phosphoinositide 3-kinase/mammalian target of rapamycin
PLCO: Prostate, lung, colorectal, and ovarian cancer screening trial
PRAD: Prostate adenocarcinoma
QTL: Quantitative trait locus
RBP: RNA binding protein
RPM: Reads per million
RPKM: Reads per kilobase per million mapped reads
RNA-seq: RNA sequencing
SENP7: SUMO specific peptidase 7
SNP: Single nucleotide polymorphism
TGCT: Testicular germ cell tumors
TCGA: The cancer genome atlas
THCA: Thyroid carcinoma
TF: Transcription factor
TIMER: Tumor immune estimation resource
TFBS: Transcription factor binding site
UKB: UK Biobank
