# Supplementary material for: Genome-wide enhancer RNA profiling adds molecular links between genetic variation and human cancers
Source: Mil Med Res. 2024 Jun 11;11:36. doi: 10.1186/s40779-024-00539-2 (PMC11165858; doi:10.1186/s40779-024-00539-2)
Supplement: Supplementary file 2 — Additional file 2: Materials and methods. Fig. S1 The three-stage studies design and workflow. Fig. S2 Overview of methods and quality control pipeline for eRNAQTL identification, integrative annotation, and validation. Fig. S3 Characterization of eRNAQTLs in TCGA 30 cancer types. Fig. S4 Pathway enrichment analyses for putative target genes of eRNAs. Fig. S5 The frequencies of amplification and deletion in eRNAQTL-eRNAs’ putative target genes. Fig. S6 Enrichment analysis on the proportion of eRNAQTL-eRNAs associated with drug response. Fig. S7 Quality assessment of the high-throughput sequence data in 10 matched colorectal cancer and normal samples. Fig. S8 Quality assessment of the RNA-sequencing data in 154 matched colorectal cancer and normal samples. Fig. S9 GO analysis for target genes of eRNAQTL-eRNAs in our CRC tissues. Fig. S10 Genomic and functional characterization of identified eRNAQTLs in colorectal cancer from TCGA dataset. Fig. S11 Characterization of eRNAQTL-eRNAs and putative target genes in colorectal cancer from TCGA dataset. Fig. S12 LD block plot (r2 ≥ 0.8) showing the r2 value of candidate variants. Fig. S13 Relative expression of ENSR00000155786 in three cell lines. Fig. S14 Effect of HOXA5 knockdown on the relative luciferase activity of vectors containing the rs3094296-T or rs3094296-C allele in SW480 and HIEC-6 cell lines. Fig. S15 The correlations between ENSR00000155786 and HOXA5 expression in all TCGA CRC samples stratified by SNP rs3094296 genotype. Fig. S16 The effect of HOXA5 knockdown on the expression level of ENSR00000155786 in HIEC-6 cells. Fig. S17 Correlations of SENP7 expression with ENSR00000155786 and HOXA5 expression in TCGA CRC samples. Fig. S18 Relative expression of SENP7 in HCT116 (rs3094296-TT) and SW480 (rs3094296-TT) cell lines compared to HCT15 (rs3094296-TC) cell line. Fig. S19 The expression level of SENP7 and ENSR00000155786 in HIEC-6 cells. Fig. S20 Differential expression of ENSR00000155786 and SENP7 in TCGA CRC [file 40779_2024_539_MOESM2_ESM.pdf]

Materials and Methods

Identification of survival-associated eRNAQTLs and GWAS-associated eRNAQTLs

Survival-associated eRNAQTLs were identified by comparing the differences in survival time between three genotype groups using a log-rank test, associations with FDR < 0.05 based on the Benjamini & Hochberg method were defined as significant. To identify GWAS-associated eRNAQTLs, all risk tag SNPs identified in GWAS studies were downloaded from the National Human Genome Research Institute (NHGRI) GWAS catalog (http://www.ebi.ac.uk/gwas/, accessed by June 2020), and GWAS linkage disequilibrium (LD) regions of these risk tag SNPs were obtained from 1000 Genomes Project. eRNAQTLs that overlapped with GWAS tag SNPs and LD SNPs ($r^2 \geq 0.5$) were defined as GWAS-associated eRNAQTLs.

Enrichment analyses of eRNAQTLs among genomic distribution

To conduct enrichment analysis, a set of control SNPs set was first generated with the distribution of minor allele frequency (MAF), number of SNPs in LD, and variant type matched to eRNAQTL SNPs among each cancer type using a web tool vSampler (version 8.0, http://mulinlab.org/vsampler/). Then genomic annotation was performed for both eRNAQTLs and control SNPs using SnpEff (version 5.1d, https://pcingola.github.io/SnpEff/) [1]. After annotation, SNPs could be classified into intronic region, untranslated region, gene upstream region, gene downstream region, splice site region, or intergenic region, and their located or nearby genes were also obtained. Enrichment analysis of eRNAQTLs among genomic annotation was performed by two-tailed Fisher's exact test with Bonferroni correction as the following 2 × 2 table: columns; eRNAQTL SNPs and control SNPs, rows; SNPs within and not within the annotated region.

Enrichment analyses of eRNAQTLs among functional annotation

The functional annotation files for histone modification ChIP-seq peaks (H3K27ac, H3K27me3, H3K4me1, H3K4me2, H3K79me2, and H3K9ac), DNase I hypersensitive sites, 801 TF-binding sites and 150 RNA binding proteins (RBPs) CLIP-seq among human cancer cell lines were downloaded from the ENCODE portal (https://www.encodeproject.org/data/annotations/). BEDtools (version 2.18, https://github.com/arq5x/bedtools2) [2] was used to find instances of SNPs overlapped with the peaks of regulatory elements. Enrichment analysis of eRNAQTLs among regulatory elements was performed by two-tailed Fisher's exact test with Bonferroni correction as the following 2 × 2 table:

columns; eRNAQTL SNPs and control SNPs, rows; SNPs within and not within the regulatory element.

Enrichment analyses of eRNAQTLs among cancer-related GWAS signals

Enrichment analyses were performed by selecting cancer-related phenotypes from NHGRI GWAS summary statistics [3] and including SNPs that reached genome-wide significance ($P < 5 \times 10^{-8}$). GWAS LD regions were defined as the genomic region containing SNPs in LD with the tag SNP at $r^2 > 0.2$. BEDtools (version 2.18, https://github.com/arq5x/bedtools2) were applied to analyze whether each SNP falls within GWAS LD regions. Enrichment analyses of eRNAQTL SNPs compared to non-eRNAQTL SNPs among cancer GWAS loci were performed by two-tailed Fisher's exact test with Bonferroni correction as the following 2 × 2 table: columns; eRNAQTL SNPs and control SNPs, rows; SNPs within and not within the disease-associated loci.

Partitioning heritability of GWAS summary statistics

To enable a more accurate comparison between the effect of eRNAQTLs on eRNA versus the effect of eQTLs on the corresponding gene expression, eQTL analysis was re-performed in each cancer type using the same TCGA cohort. The quality control and covariate adjustment pipeline were similar as above to eRNA expression data, that is, excluding genes with average expression < 0, quantile normalization, adjusting the effects of genetic principal components, PEER factors (version 1.3, https://github.com/PMBio/peer/wiki) and clinical status. A linear regression model was applied to test the association for SNPs within 1 Mb of each gene.

Then the contribution of eRNAQTLs versus eQTLs to cancer heritability was estimated by stratified LD Score Regression (S-LDSC, v1.0.1, https://github.com/bulik/ldsc) [4] using GWAS summary statistics downloaded from earlier study [5]. Enrichment for each annotation was calculated by the proportion of heritability explained by each annotation divided by the proportion of SNPs falling in that annotation category. Both separate models for each QTL annotation and a joint model with two types of QTL annotations together were analyzed, with the adjustment for various baseline annotations of SNPs using a baseline LD model, including gene annotations (coding, UTRs, intron, promoter), minor allele frequency bins and LD-related annotations. We repeated the analysis on eRNAQTLs and eQTLs at thresholds of 50%, 20%, 10%, and 5% FDR.

Putative target genes of eRNA

The heterogeneity of tumor tissues influences the analysis of clinical tumor samples by genomic approaches, including gene expression profiles. To evaluate tumor purity in tumor tissue, ESTIMATE (version 1.0.11, https://sourceforge.net/projects/estimateproject/) [6] was employed to infer stromal and immune cells in malignant tumor tissues using expression data. Then partial correlation coefficient (PCC) was calculated between the expression of eRNA and 19,430 protein-coding genes with adjustment of tumor purity. After the above calculation, the correlation coefficient between eRNA and gene was obtained, a *P*-value for the PCC, and combined to generate a rank score (RS). FDR was computed by Benjamini & Hochberg correction. Genes located within 1 Mb of eRNA regions, and the absolute correlation coefficients ≥ 0.3, FDR < 0.05 were defined as eRNA putative target genes.

Putative effects of eRNAs on signaling pathways

To analyze the biological significance of eRNA putative target genes, the Kyoto Encyclopedia of Genes and Genomes (KEGG) pathway [7] and Gene Ontology (GO) term [8] enrichment analyses were applied, and the top 10 pathways in each part were visualized. The network of enriched terms was further demonstrated by the online tool String (https://cn.string-db.org/, accessed May 2021). $P < 0.05$ was considered statistically significant.

To investigate the concrete pathways eRNAs participated in, 50 hallmark gene sets from the Molecular Signature Database (MSigDB) [9] and 17 immunologically relevant gene sets from ImmPort [10] were derived, and all co-expressed genes based on RS were ranked and subjected to gene set enrichment analysis (GSEA) [11]. All eRNA-pathway pairs with FDR < 0.05 were summarized across cancer types. Enrichment analysis of the number of eRNAQTL eRNAs among hallmark pathways was performed by a two-tailed Fisher's exact test with Bonferroni correction.

Associations between immune infiltrates and expression of eRNAs

The anticancer immune response is administered by tumor-infiltrating immune cells, thus the quantification of various types of immune infiltrates can shed light on the mechanisms underlying immunology regulation. Tumor Immune Estimation Resource (TIMER) [12] was applied to estimate the immune cell infiltration levels of each patient using gene expression profiles. The associations between immune infiltrates and eRNA expression were also evaluated by PCC with the inclusion of tumor purity as a co-variable. eRNAs with absolute correlation coefficients ≥ 0.3 and FDR < 0.05 were defined as immune infiltrates-related eRNAs. The proportions of immune-related eRNAs are

the number of eRNAQTL-eRNAs correlated with immune infiltrates divided by the total number of eRNAQTL-eRNAs.

Clinical relevance of eRNA

The multivariate Cox regression was used to assess whether eRNA expression was associated with the overall survival times of cancer patients, with the adjustment of age, sex, and tumor stage. FDR < 0.05 was considered as significant.

Associations between drug response and expression of eRNAs

Expression profile data and drug sensitivity data of human cancer cell lines were obtained from the Genomics of Drug Sensitivity in Cancer (GDSC, released June 2020, https://www.cancerrxgene.org/), which contains the sensitivity data for 198 compounds over 809 cell lines. This dataset provides the drug response result (IC_{50} values) as a measure of drug sensitivity, and lower IC_{50} values indicate increased sensitivity to treatment. OncoPredict [13] was used to impute drug response for TCGA cancer patients based on cancer molecular datasets from GDSC. Then, the Spearman's correlation was calculated between eRNA expression and predicted drug response in each cancer type, and FDR < 0.05 and absolute Spearman's correlation ≥ 0.3 were defined as significant.

Genomic variation evaluation of eRNAs-targeted genes

Masked copy number segment files that removed probes known to contain germline mutations were downloaded from the TCGA data portal and significant focal copy number alterations were determined from these segmented data using GISTIC 2.0 (version 2.0.22, https://broadinstitute.github.io/gistic2/) [14]. For each locus, a sample is called deep amplification if the value is +2 (i.e., higher than the maximum of these arm values), while a −2 (deep deletion) is a value less than the minimum of these values. Shallow (±1) amplifications and deletions correspond to alterations between 0.1 relative copy number and the thresholds for deep alterations. Masked somatic mutation calls identified by the MuTect2 pipeline are downloaded from the TCGA data portal, which detected not only somatic single-nucleotide variations but also small insertions and deletions. TMB was calculated by summing the total number of all mutations and dividing by the size of the captured exome (50 Mb).

After estimating the percentage of genome that was affected by copy number gains (the fraction of amplified genome) or losses (the fraction of deleted genome), and the number of non-silent

mutations for each sample, a two-sided Student's *t*-test was performed to evaluate the difference between target genes of eRNA with or without eRNAQTL in two molecular characteristics (copy number variation and non-silent mutations). Visualization of a heatmap of copy number variation and mutation landscape was conducted by the R package ComplexHeatmap and maftools.

eRNA and mRNA analysis in Chinese CRC tissues

ATAC-seq and analysis

ATAC-seq was performed by SeqHealth (Wuhan, China). About 500 mg tissue was treated with cold ATAC lysis buffer and the nucleus was collected by centrifuging for 10 min at 500 *g*. Transposition and high-throughput DNA sequencing library were carried out by TruePrep DNA Library Prep Kit V2 for Illumina kit (Vazyme, China). The library products were enriched, quantified, and finally sequenced on a Novaseq 6000 sequencer (Illumina, California, USA) using the PE150 model. Raw sequencing data was first filtered by Trimmomatic (version 0.36, https://github.com/usadellab/Trimmomatic) to remove low-quality reads and adapters. Clean reads were further treated with FastUniq (version 1.1, http://sourceforge.net/projects/fastuniq/) to eliminate duplication. Then deduplicated reads were mapped to the human reference genome using Bowtie2 (version 2.2.6, https://bowtie-bio.sourceforge.net/bowtie2/index.shtml) with default parameters. Afterward, peak calling and peak annotation were conducted using the MACS2 software (version 2.2.9.1, https://pypi.org/project/MACS2/) and BEDtools (version 2.18, https://github.com/arq5x/bedtools2), respectively.

H3K27ac ChIP-seq assay and analysis

ChIP-seq assays were performed with Magna ChIP™ G Tissue Kit (Millipore, USA). As described previously [15], tissue was fixed in 1% formaldehyde for 10 min at room temperature, and quenched with 0.125 mol/L glycine for 5 min to terminate the crosslinking reaction. Then the tissue was disaggregated with cell lysis buffer and the nucleus was collected by centrifuging at 800 *g* for 5 min at 4 °C. Next, the nucleus was treated with nucleus lysis buffer, and sonication was performed to break chromatin into fragments. For immunoprecipitation reactions, antibodies against H3K27ac (CREam, USA) and a nonspecific rabbit IgG (Santa Cruz, USA) were incubated overnight with the crosslinked protein and DNA, followed by adding protein A/G magnetic beads. DNA fragments were purified and collected by a Dr.GenTLE Precipitation Carrier kit (TaKaRa, Japan). Then, the purified

DNA library was sequenced on a Novaseq 6000 sequencer (Illumina) with a PE150 model by SeqHealth (Wuhan, China). Afterward, quality control, human genome mapping, and peak calling were performed as described in ATAC-seq analysis.

RNA-seq and analysis

RNA-seq was conducted as previously described [15]. Total RNAs were extracted from frozen tissue using TRIzol (Invitrogen, USA). Total amounts and integrity of RNA were assessed using the RNA Nano 6000 Assay Kit of the Bioanalyzer 2100 system (Agilent Technologies, CA, USA). For library preparation, the Illumina RNA Library Prep kit (NEBNext® UltraTM) was used. Libraries were tagged with unique adapter indexes. Final libraries were validated on the Agilent 2100 Bioanalyzer, quantified via qRT-PCR, pooled at equimolar ratios, diluted, denatured, and loaded onto an Illumina novaseq 6000. Clean data (clean reads) were obtained by removing reads containing adapter, reads containing N base and low-quality reads from raw data. At the same time, Q20, Q30, and GC content in the clean data were calculated, which are summarized in **Additional file 2: Fig. S8**. All the downstream analyses were based on the clean data with high quality. Trimmed reads were aligned to human transcriptome using the Genome Reference Consortium human reference 37 assembly (GRCh37/hg19) with Hisat (version 2.0.5, https://ccb.jhu.edu/software/hisat/index.shtml) software. Only samples with a depth of coverage greater than 10 million mappable paired-end reads, a multimapping rate lower than 10%, and a unique mapping rate greater than 60% remained. The mean library size was 6 G (> 5.88 G). Gene and transcript expression were quantified with featureCounts.

Differential expression analysis in Chinese CRC tissues

Only genes whose average expression value (count ≥ 1) in normal and tumor samples were considered for analysis. After the raw gene count was normalized, the DEseq R package (version 2.10, https://bioconductor.org/packages//2.10/bioc/html/DESeq.html) [16] was used to calculate differentially expressed genes. The resulting *P*-values were adjusted using Benjamini and Hochberg’s approach for controlling the false discovery rate. Genes with more than two-fold change and *q*-value < 0.05 were assigned as differentially expressed.

The comparison and characterization of eRNAQTLs in CRC tumor and normal tissues

For quantifying eRNAQTL sharing among tumor and normal tissue in a continuous way, the R package *q*-value (version 2.34.0, https://bioconductor.org/packages/qvalue) [17] was applied with

default recommended settings. This program takes a list of *P*-values and computes their estimated $\pi0$ (the proportion of features that are truly null) based on their distribution. The quantity $\pi1 = 1 - \pi0$ estimates the lower bound of true positive associations. Sharing signals between two tissues is calculated as the $\pi1$ estimated from the *P*-value distribution of the overlapping SNP-eRNA pairs in each tissue. For the sharing between CRC GWAS data and eRNAQTLs, these *P*-values were overlapped with normal-specific, tumor-specific, and shared eRNAQTLs. From the resulting GWAS *P*-value distributions, the $\pi1$ statistic was calculated using bootstrapping, signifying the proportion of estimated true positives in the distribution.

Study populations and phenotype definitions

Chinese population in three-stage GWASs

In the discovery stage, 968 CRC patients and 1321 controls were recruited from the Chinese Academy of Medical Sciences in Beijing, China; 3325 patients and 5855 controls were recruited from Tongji Hospital of Huazhong University of Science and Technology, Zhongnan Hospital of Wuhan University, and Renmin Hospital of Wuhan University in Wuhan, China. Primary CRC was confirmed by histopathological or cytological examination according to the World Health Organization classification. All controls were healthy individuals selected from routine physical examinations in the same region during the same period when patients were recruited. Peripheral blood samples and demographic characteristics including sex, age, smoking status, and drinking status were obtained from the medical records and interviews at recruitment. Demographic characteristics could be found in our previous study [18].

The replication I phase recruited 1524 CRC cases and 1522 cancer-free controls from the cancer hospital of the Chinese Academy of Medical Sciences in Beijing, China. The replication II phase included 4500 cases and 8500 cancer-free controls from Tongji Hospital of Huazhong University of Science and Technology (Wuhan, China). The inclusion/exclusion criteria are the same as the first discovery stage. Demographic characteristics could be found in our previous study [19].

European populations in three-stage GWASs

For another independent three-stage GWAS in the European population, we adopted three datasets. The discovery stage was conducted in the Genetics and Epidemiology of Colorectal Cancer Consortium (GECCO) [20], which encompassed 19,951 controls and 17,789 CRC patients. The

demographic characteristics could be found in our previous study [18].

Furthermore, the results were replicated in two independent European datasets. Phase I was conducted in Prostate, Lung, Colorectal, and Ovarian Cancer Screening Trial (PLCO) [21]. PLCO enrolled 154,934 participants (men and women between 55 and 74 years of age) at 10 centers in a large, randomized, two-arm trial to determine the effectiveness of screening to reduce cancer mortality. Details of this study have been previously described and are available online (http://dcp.cancer.gov/plco). After excluding patients with other cancers according to self-reported and questionnaire data, 1233 primary invasive colorectal cancer cases diagnosed during the trial were included. A total of 6165 controls were randomly selected and frequency-matched to cases at the 5:1 ratio. Matching criteria were age at enrollment (two-year blocks) and gender.

In phase II, genotype data was adopted from the UK Biobank (UKB) [22], a prospective cohort study that recruited over 500,000 participants aged between 37 and 73 years from the general population from 2006 to 2010. The study design, recruitment, cohort profile, and data collection have been described in detail at UKB (http://www.ukbiobank.ac.uk/). Cases were defined as subjects with primary invasive colorectal cancer based on the 10th Revision of the International Classification of Diseases (ICD10, C18, C19, C20, except for C18.1). A total of 5246 patients diagnosed with CRC as a first primary malignancy were included. For each case, we selected 5 eligible controls from cancer-free subjects with enrollment age and gender as matching criteria, resulting in 26,230 controls being enrolled.

Genotyping, quality control and imputation in Chinese populations

In the discovery stage, subjects were genotyped on two platforms. Genome-wide scanning for 968 CRC cases and 1321 controls from Beijing was conducted using the IlluminaTM Global Screening Array (GSA) system. After quality control, 5,928,562 SNPs remained for association analysis. Genotyping for 3333 cases and 5855 controls from Wuhan was performed using the IlluminaTM Asian Screening Array (ASA) system. After quality control, 7,768,486 SNPs were remained for association analysis. The detailed process of quality control has been described in our previous study [18].

In the two replication stages, SNP rs3094296 was genotyped in 6024 CRC cases and 10,022 controls using the TaqMan assays platform (ABI 7900HT system, Applied Biosystems). Quality control was implemented as described previously [19].

Genotyping, quality control and imputation in European populations

In the discovery stage, GECCO genotype data was downloaded from the database of Genotypes and Phenotypes (dbGaP) under accession number phs001078.v1.p1, phs001315.v1.p1 and phs001415.v1.p1. Imputation was conducted using the Michigan Imputation Server with Haplotype Reference Consortium r1.1.2016 (HRC) as a reference panel. All batches were merged into a single set after imputation and excluded SNPs as following criteria: 1) SNPs with imputation quality < 0.4; 2) SNPs with MAF < 1%; 3) SNPs deviating from the Hardy-Weinberg equilibrium ($P < 10^{-6}$); 4) SNPs with missing call frequencies > 0.02 and SNPs located in the sex chromosome. Additionally, samples without age information were removed. A total of 19,951 controls and 17,789 CRC patients with 6,263,205 SNPs have finally remained.

In the replication I stage, genotype data for PLCO was obtained from dbGaP under accession number phs000346.v2.p2, phs001554.v1.p1, phs001286.v2.p2, and phs001524.v1.p1. Genotyping was completed using Illumina HumanHap300, HumanHap240S, and 610K BeadChip Array System on the Infinium platform, and has been described previously [21]. Genotype data for SNP rs3094296 in 1233 CRC cases and 6165 controls was extracted for GWAS analysis.

In the replication II stage, genotype data imputed to the HRC.r1-1 panel was obtained from UKB (http://www.ukbiobank.ac.uk/) under Application No. 94939. Genotyping, imputation, and quality control were described elsewhere [22]. Genotype data for SNP rs3094296 in 5246 CRC cases and 26,230 controls was extracted for GWAS analysis.

References

1. Cingolani P, Platts A, Wang Le L, Coon M, Nguyen T, Wang L, et al. A program for annotating and predicting the effects of single nucleotide polymorphisms, SnpEff: SNPs in the genome of Drosophila melanogaster strain w1118; iso-2; iso-3. Fly (Austin). 2012;6(2):80-92.

2. Quinlan AR, Hall IM. BEDTools: a flexible suite of utilities for comparing genomic features. Bioinformatics. 2010;26(6):841-2.

3. Buniello A, Macarthur JAL, Cerezo M, Harris LW, Hayhurst J, Malangone C, et al. The NHGRI-EBI GWAS catalog of published genome-wide association studies, targeted arrays and summary statistics 2019. Nucleic Acids Res. 2019;47(D1):D1005-D12.

4. Finucane HK, Bulik-Sullivan B, Gusev A, Trynka G, Reshef Y, Loh PR, et al. Partitioning heritability by functional annotation using genome-wide association summary statistics. Nat Genet. 2015;47(11):1228-35.

5. Rashkin SR, Graff RE, Kachuri L, Thai KK, Alexeeff SE, Blatchins MA, et al. Pan-cancer study detects genetic risk variants and shared genetic basis in two large cohorts. Nat Commun. 2020;11(1):4423.

6. Yoshihara K, Shahmoradgoli M, Martinez E, Vegesna R, Kim H, Torres-Garcia W, et al. Inferring tumour purity and stromal and immune cell admixture from expression data. Nat Commun. 2013;4:2612.

7. Kanehisa M, Goto S. KEGG: kyoto encyclopedia of genes and genomes. Nucleic Acids Res. 2000;28(1):27-30.

8. Gene Ontology C. Gene Ontology Consortium: going forward. Nucleic Acids Res. 2015;43(Database issue):D1049-D56.

9. Liberzon A, Birger C, Thorvaldsdottir H, Ghandi M, Mesirov JP, Tamayo P. The Molecular Signatures Database (MSigDB) hallmark gene set collection. Cell Syst. 2015;1(6):417-25.

10. Bhattacharya S, Andorf S, Gomes L, Dunn P, Schaefer H, Pontius J, et al. ImmPort: disseminating data to the public for the future of immunology. Immunol Res. 2014;58(2-3):234-9.

11. Subramanian A, Tamayo P, Mootha VK, Mukherjee S, Ebert BL, Gillette MA, et al. Gene set enrichment analysis: a knowledge-based approach for interpreting genome-wide expression profiles. Proc Natl Acad Sci U S A. 2005;102(43):15545-50.

12. Li T, Fan J, Wang B, Traugh N, Chen Q, Liu JS, et al. TIMER: a web server for comprehensive analysis of tumor-infiltrating immune cells. Cancer Res. 2017;77(21):e108-e10.

13. Maeser D, Gruener RF, Huang RS. oncoPredict: an R package for predicting in vivo or cancer patient drug response and biomarkers from cell line screening data. Brief Bioinform. 2021;22(6):bbab260.

14. Mermel CH, Schumacher SE, Hill B, Meyerson ML, Beroukhim R, Getz G. GISTIC2.0 facilitates sensitive and confident localization of the targets of focal somatic copy-number alteration in human cancers. Genome Biol. 2011;12(4):R41.

15. Tian J, Chang J, Gong J, Lou J, Fu M, Li J, et al. Systematic functional interrogation of genes in GWAS loci identified ATF1 as a key driver in colorectal cancer modulated by a promoter-enhancer interaction. Am J Hum Genet. 2019;105(1):29-47.

16. Anders S, Huber W. Differential expression analysis for sequence count data. Genome Biol. 2010;11(10):R106.

17. Storey JD, Tibshirani R. Statistical significance for genomewide studies. Proc Natl Acad Sci U S A. 2003;100(16):9440-5.

18. Zhang M, Chen C, Lu Z, Cai Y, Li Y, Zhang F, et al. Genetic control of alternative splicing and its distinct role in colorectal cancer mechanisms. Gastroenterology. 2023;165(5):1151-67.

19. Tian J, Lou J, Cai Y, Rao M, Lu Z, Zhu Y, et al. Risk SNP-mediated enhancer-promoter interaction drives colorectal cancer through both FADS2 and AP002754.2. Cancer Res. 2020;80(9):1804-18.

20. Huyghe JR, Bien SA, Harrison TA, Kang HM, Chen S, Schmit SL, et al. Discovery of common and rare genetic risk variants for colorectal cancer. Nat Genet. 2019;51(1):76-87.

21. Peters U, Hutter CM, Hsu L, Schumacher FR, Conti DV, Carlson CS, et al. Meta-analysis of new genome-wide association studies of colorectal cancer risk. Hum Genet. 2012;131(2):217-34.

22. Bycroft C, Freeman C, Petkova D, Band G, Elliott LT, Sharp K, et al. The UK Biobank resource with deep phenotyping and genomic data. Nature. 2018;562(7726):203-9.

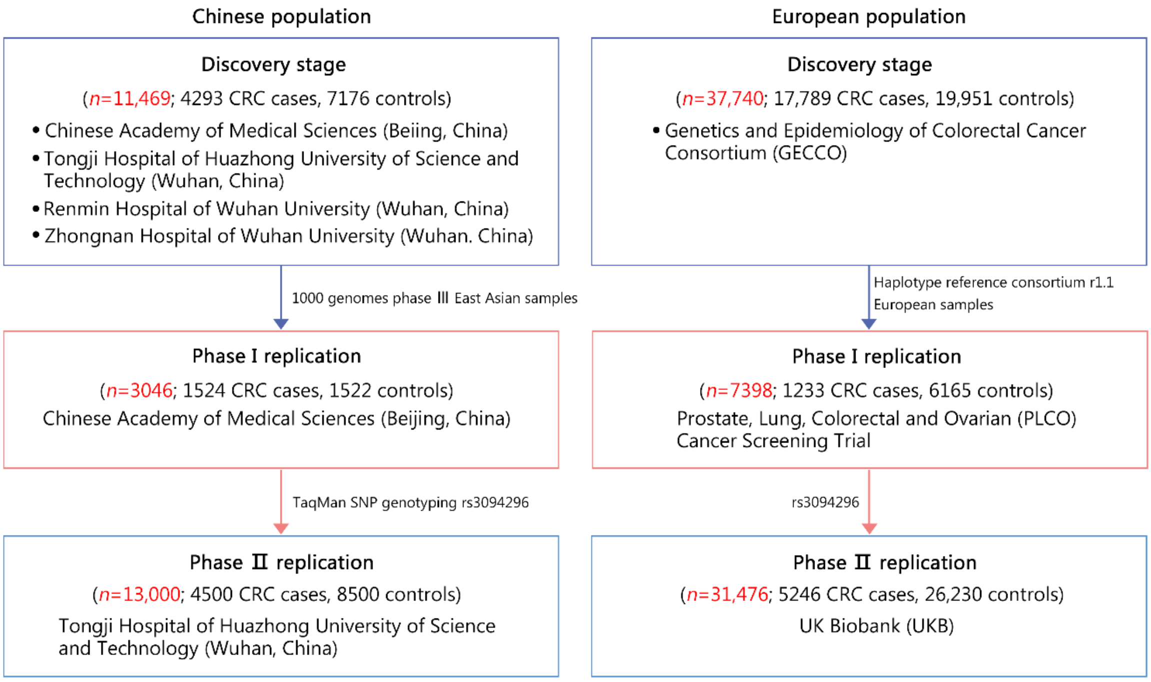

Fig. S1 The three-stage studies design and workflow. The discovery stage involved 4293 CRC patients and 7176 controls from the Chinese population 17,789 CRC patients and 19,951 controls from the European population. In the two replication stages, rs3094296 was validated in 12,503 cases and 42,417 controls. CRC colorectal cancer, SNP single nucleotide polymorphism

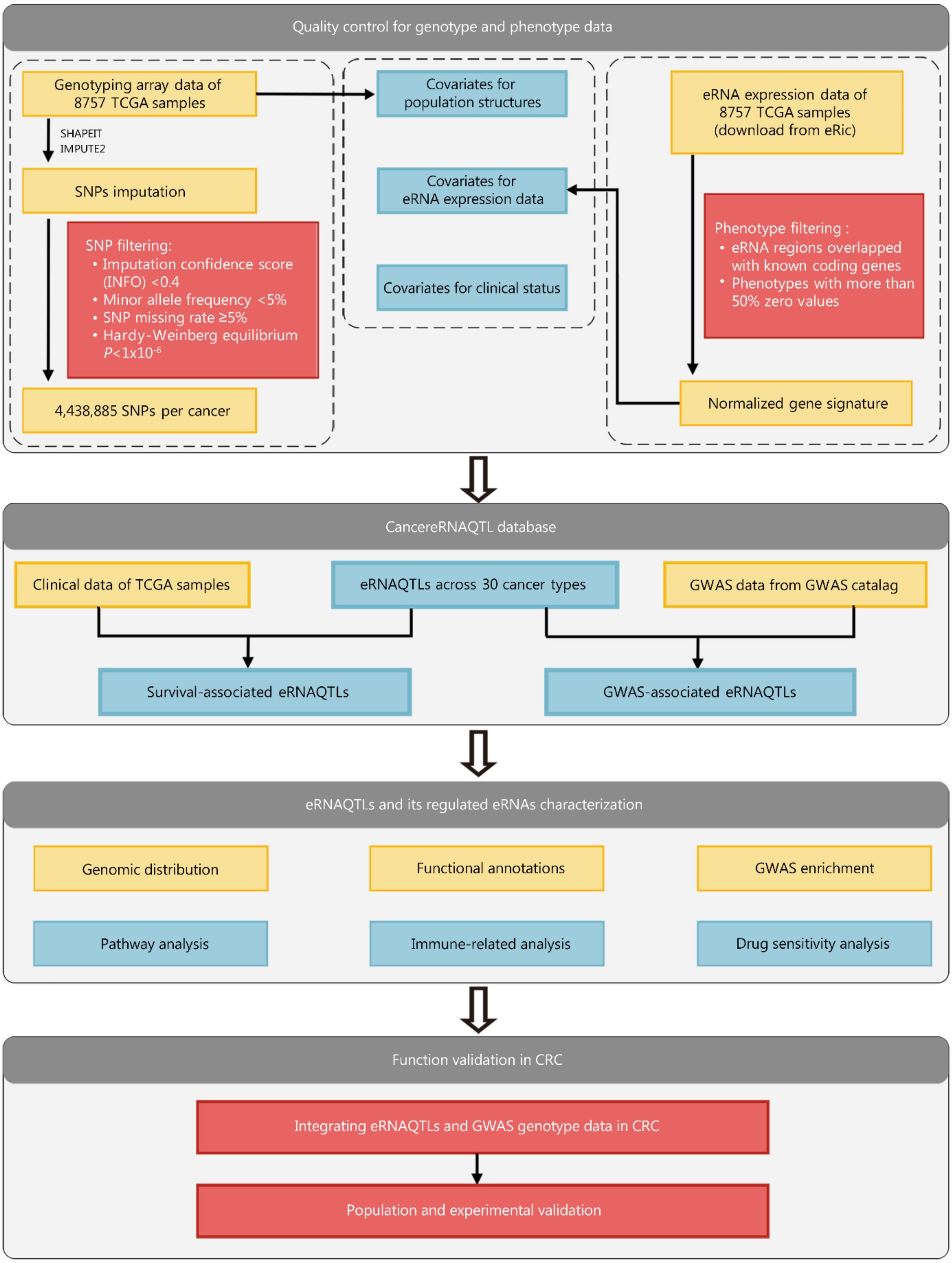

Fig. S2 Overview of methods and quality control pipeline for eRNAQTL identification, integrative annotation, and validation. TCGA The Cancer Genome Atlas, SNPs single nucleotide polymorphisms, GWAS genome-wide association study, CRC colorectal cancer, eRNAQTL enhancer RNA quantitative trait locus

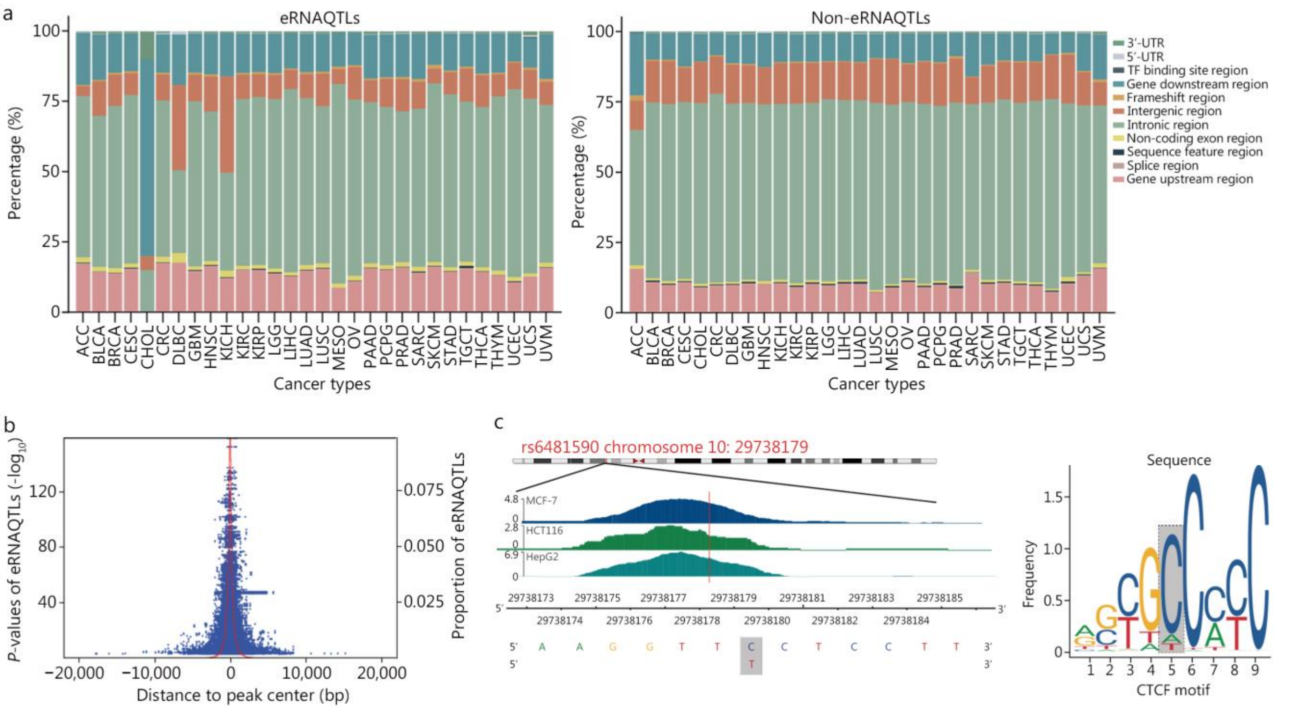

Fig. S3 Characterization of eRNAQTLs in TCGA 30 cancer types. **a** Genomic distribution of eRNAQTLs and matched control SNPs. Control SNPs are selected based on matching the number of variants in LD, minor allele frequency, and variant type. **b** Probability distribution density profile. Each blue dot indicates an SNP plotted according to its distance to the nearest H3K4me1 peak center (known enhancer marker) and statistical significance for association with eRNAs expression (-$\log_{10}$ *P*-value). The red line indicates the proportion of SNPs (%) that were classified as eRNAQTL SNPs. **c** An example of eRNAQTL overlaps the CTCF ChIP-seq peak in MCF-7, HCT116, and HepG2 cells (top), rs6481590-C allele resides within CTCF binding motif predicted from JASPAR (bottom). ACC adrenocortical carcinoma, BLCA bladder urothelial carcinoma, BRCA breast invasive carcinoma, CESC cervical squamous cell carcinoma and endocervical adenocarcinoma, CHOL cholangiocarcinoma, CRC colorectal cancer, DLBC lymphoid neoplasm diffuses large B-cell lymphoma, GBM glioblastoma multiforme, HNSC head and neck squamous cell carcinoma, KICH kidney chromophobe, KIRC kidney renal clear cell carcinoma, KIRP kidney renal papillary cell carcinoma, LGG lower grade glioma, LIHC liver hepatocellular carcinoma, LUAD lung adenocarcinoma, LUSC lung squamous cell carcinoma, MESO mesothelioma, OV ovarian serous cystadenocarcinoma, PAAD pancreatic adenocarcinoma, PCPG pheochromocytoma and paraganglioma, PRAD prostate adenocarcinoma, SARC sarcoma, SKCM skin cutaneous melanoma, STAD stomach adenocarcinoma, TGCT testicular germ cell tumors, THCA thyroid carcinoma,

THYM thymoma, UCEC uterine corpus endometrial carcinoma, UCS uterine carcinosarcoma, UVM uveal melanoma, 3’-UTR 3’ untranslated region, 5’-UTR 5’ untranslated region, TF transcription factor, SNP single nucleotide polymorphism, bp base pair, CTCF CCCTC-binding factor, JASPAR joint analysis of sequence profiles for unbiased recognition of transcription factor binding sites, TCGA The Cancer Genome Atlas, eRNAQTLs eRNA quantitative trait loci, LD linkage disequilibrium, ChIP-seq chromatin immunoprecipitation sequencing

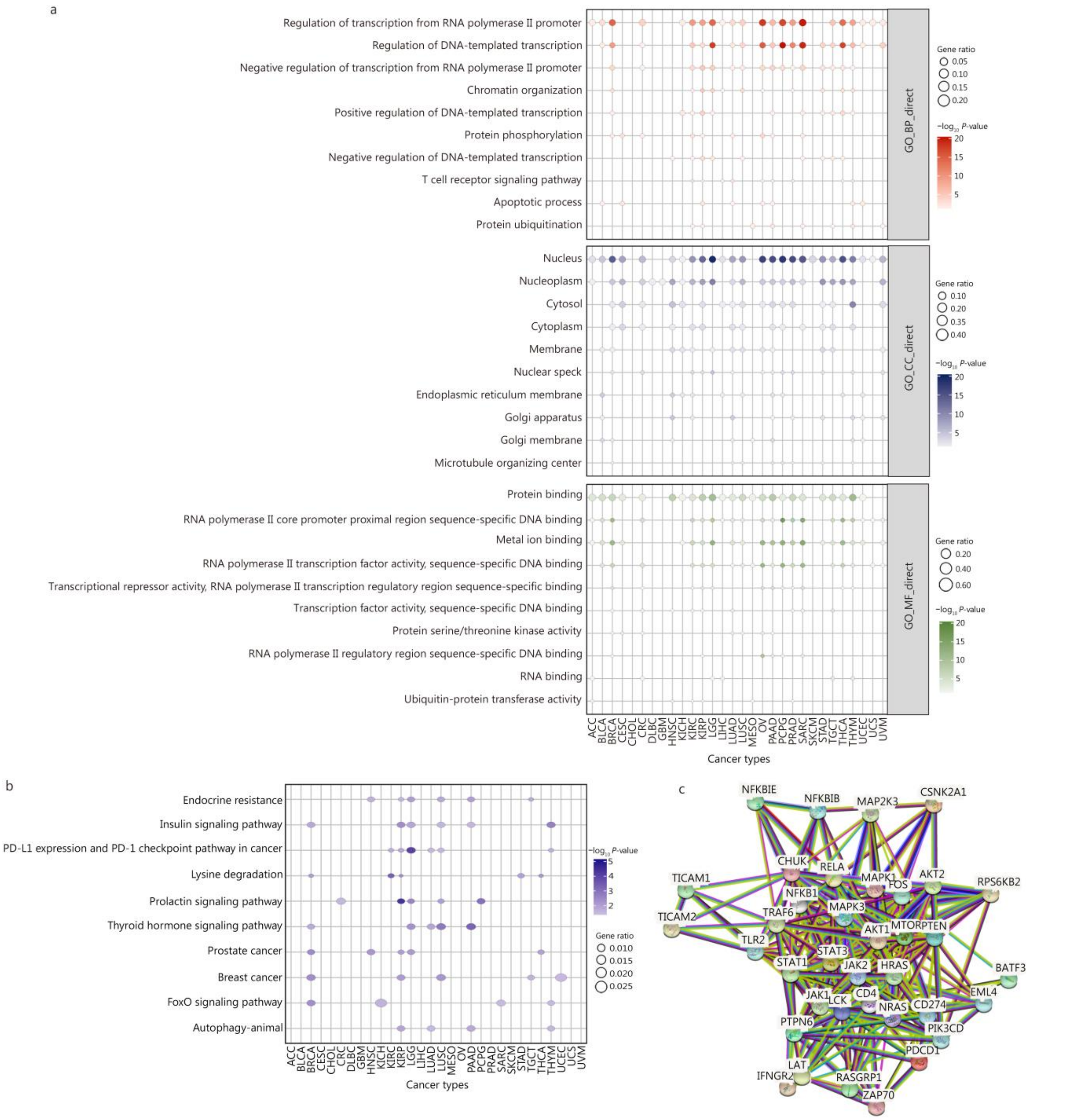

Fig. S4 Pathway enrichment analyses for putative target genes of eRNAs. **a** GO term enrichment analyses for putative target genes of eRNAQTL-eRNAs, only the top 10 significant pathways among BP, CC, and MF are visualized. The circle color represents the -$\log_{10}$ *P*-value, circle size represents the ratio of gene count. **b** KEGG enrichment analyses for putative target genes of eRNAQTL-eRNAs, only the top 10 significant pathways are listed. The circle color represents the -$\log_{10}$ *P*-value, circle size represents the ratio of gene count. **c** Visualization of PD-L1 expression and PD-1 checkpoint pathway in cancer network, by online tool String. eRNAQTL eRNA quantitative trait locus, GO Gene Ontology, BP biological process, CC cell component, MF molecular function, KEGG Kyoto

Encyclopedia of Genes and Genomes, ACC adrenocortical carcinoma, BLCA bladder urothelial carcinoma, BRCA breast invasive carcinoma, CESC cervical squamous cell carcinoma and endocervical adenocarcinoma, CHOL cholangiocarcinoma, CRC colon and rectum adenocarcinoma, DLBC lymphoid neoplasm diffuses large B-cell lymphoma, GBM glioblastoma multiforme, HNSC head and neck squamous cell carcinoma, KICH kidney chromophobe, KIRC kidney renal clear cell carcinoma, KIRP kidney renal papillary cell carcinoma, LGG lower grade glioma, LIHC liver hepatocellular carcinoma, LUAD lung adenocarcinoma, LUSC lung squamous cell carcinoma, MESO mesothelioma, OV ovarian serous cystadenocarcinoma, PAAD pancreatic adenocarcinoma, PCPG pheochromocytoma and paraganglioma, PRAD prostate adenocarcinoma, SARC sarcoma, SKCM skin cutaneous melanoma, STAD stomach adenocarcinoma, TGCT testicular germ cell tumors, THCA thyroid carcinoma, THYM thymoma, UCEC uterine corpus endometrial carcinoma, UCS uterine carcinosarcoma, UVM uveal melanoma, PD-1 programmed death 1, PD-L1 programmed death ligand 1, FoxO forkhead box O

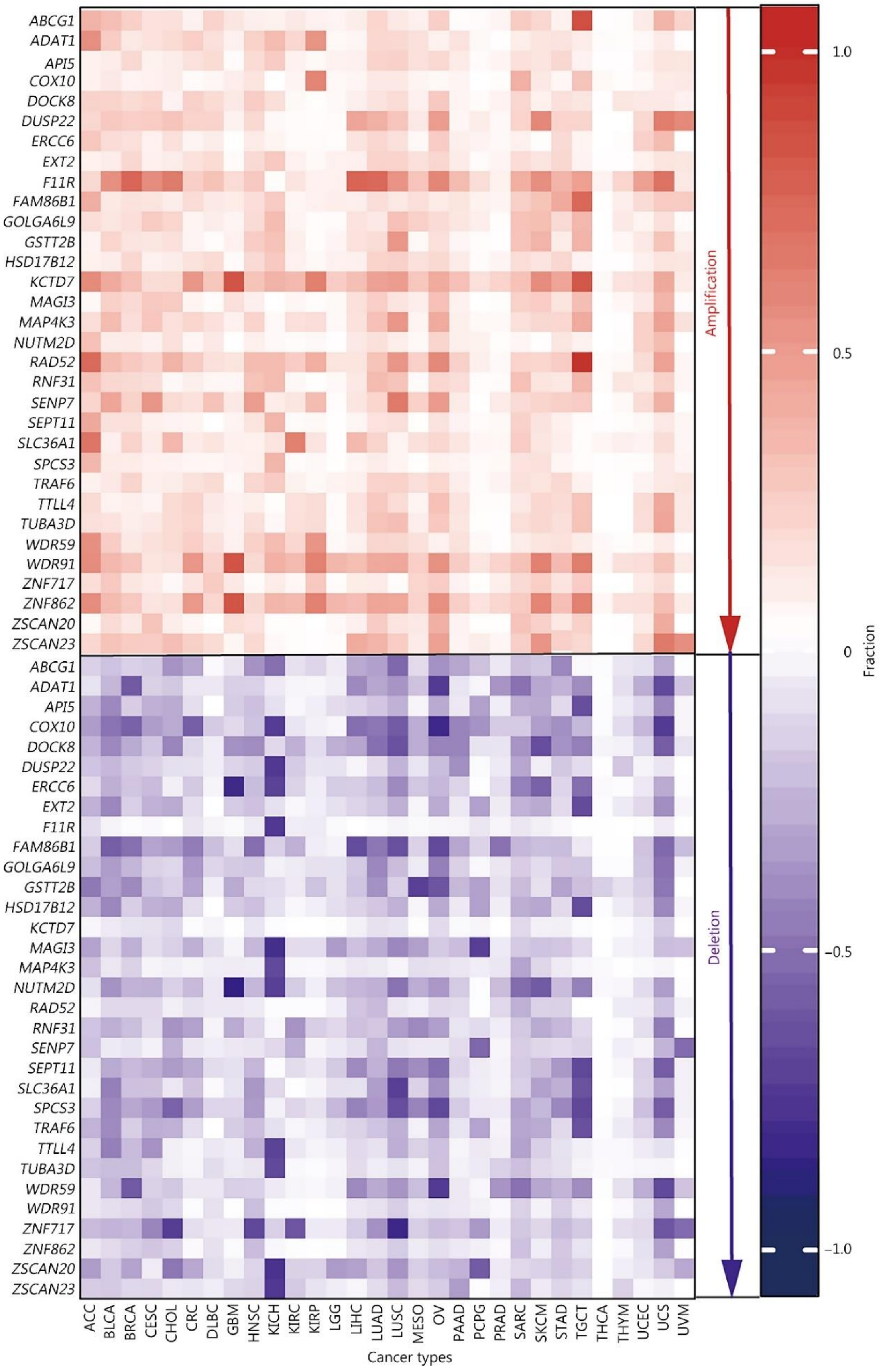

Fig. S5 The frequencies of amplification and deletion in eRNAQTL-eRNAs' putative target genes. Genes recurrently mutated among 30 cancer types were only shown. Red indicates amplifications

and blue indicates deletions. ABCG1 ATP-binding cassette sub-family G member 1, ADAT1 adenosine deaminase tRNA-specific 1, API5 apoptosis inhibitor 5, COX10 cytochrome C oxidase assembly factor 10, DOCK8 dedicator of cytokinesis 8, DUSP22 dual specificity phosphatase, ERCC6 excision repair 6, EXT2 exostosin glycosyltransferase, F11R F11 receptor, FAM86B1 family with sequence similarity 86 member B1, GOLGA6L9 golgin A6 family like 9, GSTT2B glutathione s-transferase theta 2B, HSD17B12 hydroxysteroid 17-beta dehydrogenase 12, KCTD7 potassium channel tetramerization domain containing 7, MAGI3 membrane associated guanylate kinase, MAP4K3, mitogen-activated protein kinase 3, RAD52 radiation sensitive 52, RNF31 ring finger protein 31, SENP7 sentrin-specific protease 7, SEPT11 septin 11, SLC36A1 solute carrier family 36 member 1, SPCS3 signal peptidase complex subunit 3, TRAF6 tumor necrosis factor receptor associated factor 6, TTLL4 tubulin tyrosine ligase like 4, TUBA3D tubulin alpha 3D, WDR59 WD repeat domain 59, WDR91 WD repeat domain 91, ZNF717 zinc finger protein 717, ZNF862 zinc finger protein 862, ZSCAN20 zinc finger and SCAN domain containing 20, ZSCAN23 zinc finger and SCAN domain containing 23, ACC adrenocortical carcinoma, BLCA bladder urothelial carcinoma, BRCA breast invasive carcinoma, CESC cervical squamous cell carcinoma and endocervical adenocarcinoma, CHOL cholangiocarcinoma, CRC colon and rectum adenocarcinoma, DLBC lymphoid neoplasm diffuses large B-cell lymphoma, GBM glioblastoma multiforme, HNSC head and neck squamous cell carcinoma, KICH kidney chromophobe, KIRC kidney renal clear cell carcinoma, KIRP kidney renal papillary cell carcinoma, LGG lower grade glioma, LIHC liver hepatocellular carcinoma, LUAD lung adenocarcinoma, LUSC lung squamous cell carcinoma, MESO mesothelioma, OV ovarian serous cystadenocarcinoma, PAAD pancreatic adenocarcinoma, PCPG pheochromocytoma and paraganglioma, PRAD prostate adenocarcinoma, SARC sarcoma, SKCM skin cutaneous melanoma, STAD stomach adenocarcinoma, TGCT testicular germ cell tumors, THCA thyroid carcinoma, THYM thymoma, UCEC uterine corpus endometrial carcinoma, UCS uterine carcinosarcoma, UVM uveal melanoma

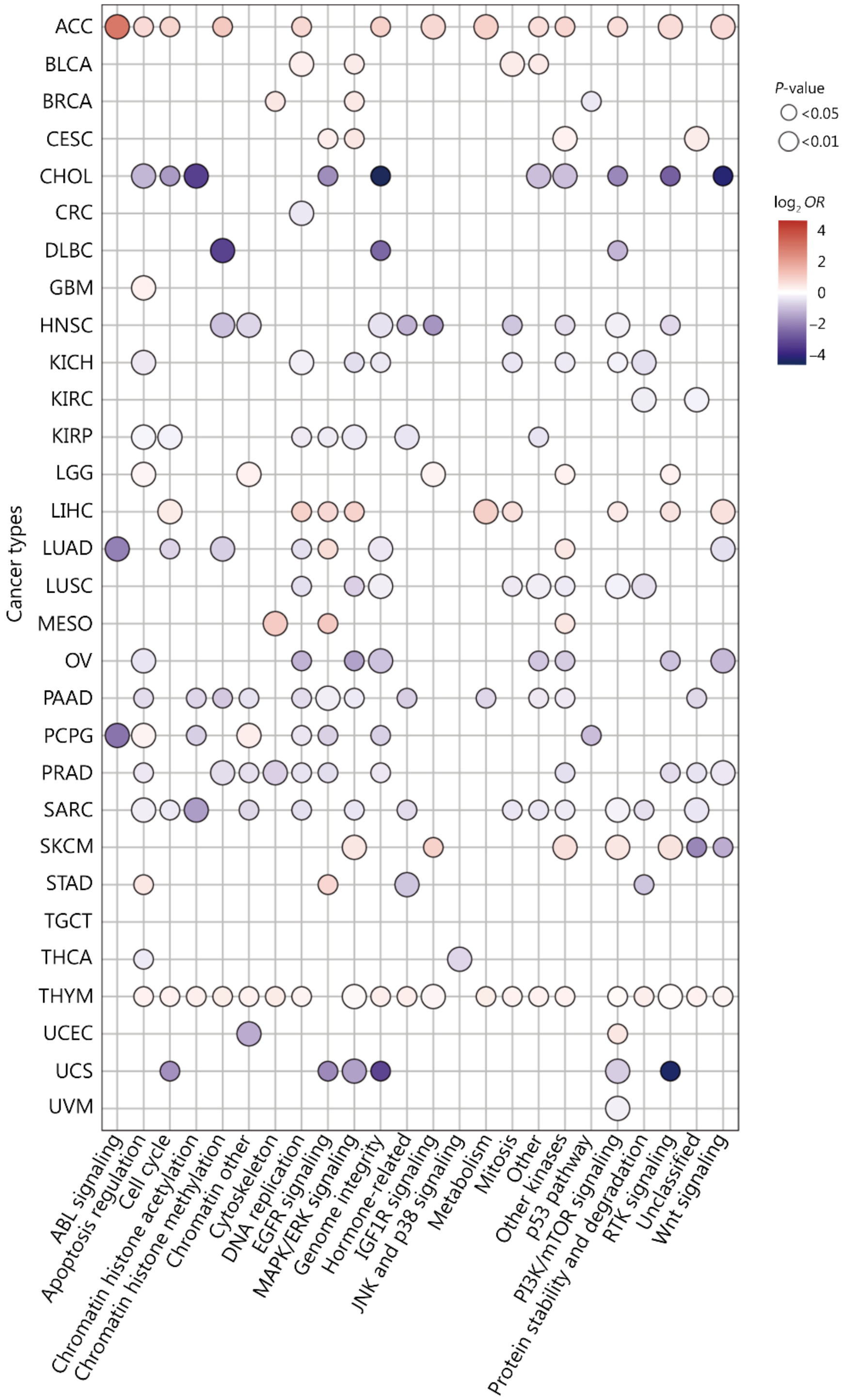

Fig. S6 Enrichment analysis on the proportion of eRNAQTL-eRNAs associated with drug response. The drug response was predicted based on the Genomics of Drug Sensitivity in Cancer (GDSC) dataset. ACC adrenocortical carcinoma, BLCA bladder urothelial carcinoma, BRCA breast invasive carcinoma, CESC cervical squamous cell carcinoma and endocervical adenocarcinoma, CHOL cholangiocarcinoma, CRC colon and rectum adenocarcinoma, DLBC lymphoid neoplasm diffuses large B-cell lymphoma, GBM glioblastoma multiforme, HNSC head and neck squamous cell

carcinoma, KICH kidney chromophobe, KIRC kidney renal clear cell carcinoma, KIRP kidney renal papillary cell carcinoma, LGG lower grade glioma, LIHC liver hepatocellular carcinoma, LUAD lung adenocarcinoma, LUSC lung squamous cell carcinoma, MESO mesothelioma, OV ovarian serous cystadenocarcinoma, PAAD pancreatic adenocarcinoma, PCPG pheochromocytoma and paraganglioma, PRAD prostate adenocarcinoma, SARC sarcoma, SKCM skin cutaneous melanoma, STAD stomach adenocarcinoma, TGCT testicular germ cell tumors, THCA thyroid carcinoma, THYM thymoma, UCEC uterine corpus endometrial carcinoma, UCS uterine carcinosarcoma, UVM uveal melanoma, WNT wingless-related integration site, RTK receptor tyrosine kinase, PI3K/mTOR phosphoinositide 3-kinase/mammalian target of rapamycin pathway, JNK and p38 Jun N-terminal kinase and p38 mitogen-activated protein kinase, IGF1R insulin-like growth factor 1 receptor, MAPK/ERK mitogen-activated protein kinase/extracellular signal-regulated kinase, EGFR epidermal growth factor receptor, ABL abelson

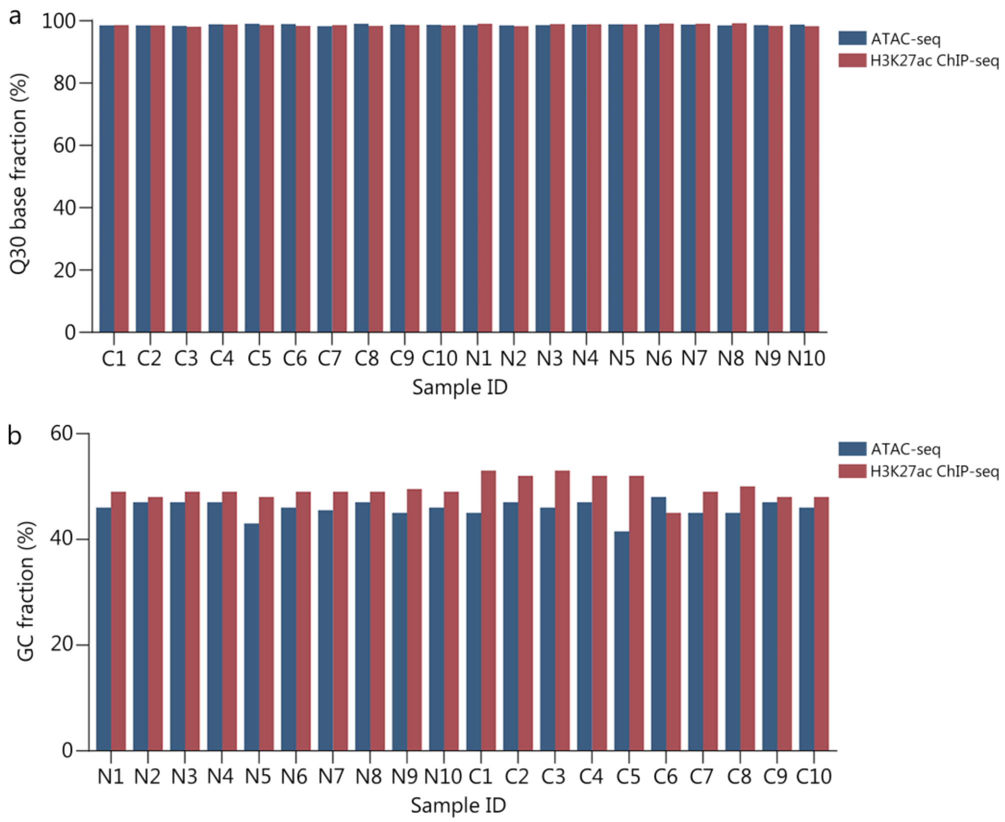

Fig. S7 Quality assessment of the high-throughput sequence data in 10 matched colorectal cancer and normal samples. **a** The fraction of the sequenced bases with a quality score of at least Q30. **b** The fraction of the GC content. Q30 sequencing quality, GC guanine cytosine, ATAC-seq assay for transposase-accessible chromatin with high throughput sequencing, ChIP-seq chromatin immunoprecipitation sequencing, Q30 sequencing quality, GC guanine cytosine

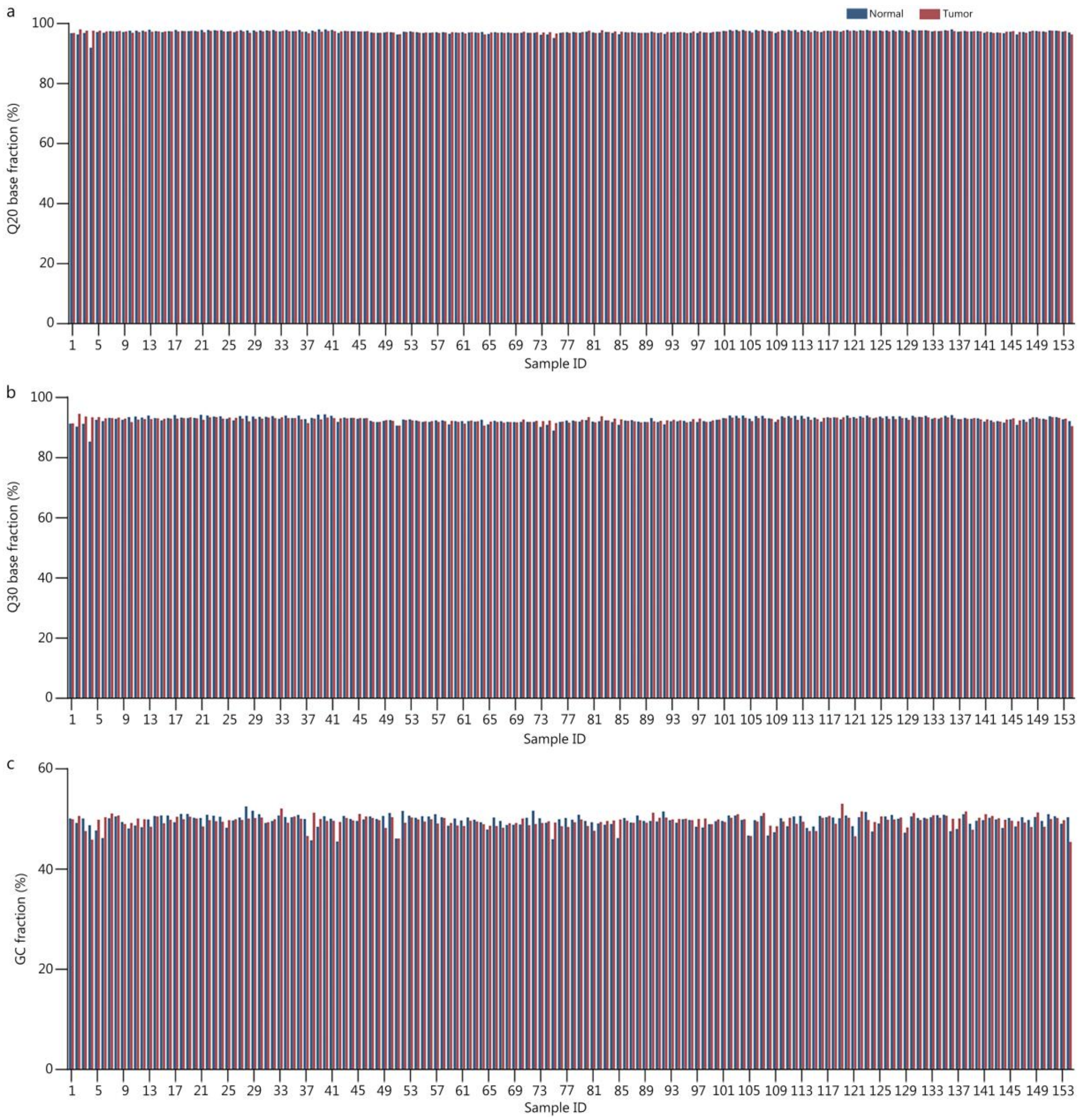

Fig. S8 Quality assessment of the RNA-sequencing data in 154 matched colorectal cancer and normal samples. **a** The fraction of the sequenced bases with a quality score of at least Q20. **b** The fraction of the sequenced bases with a quality score of at least Q30. **c** The fraction of the GC content. Q30 sequencing quality, GC guanine cytosine

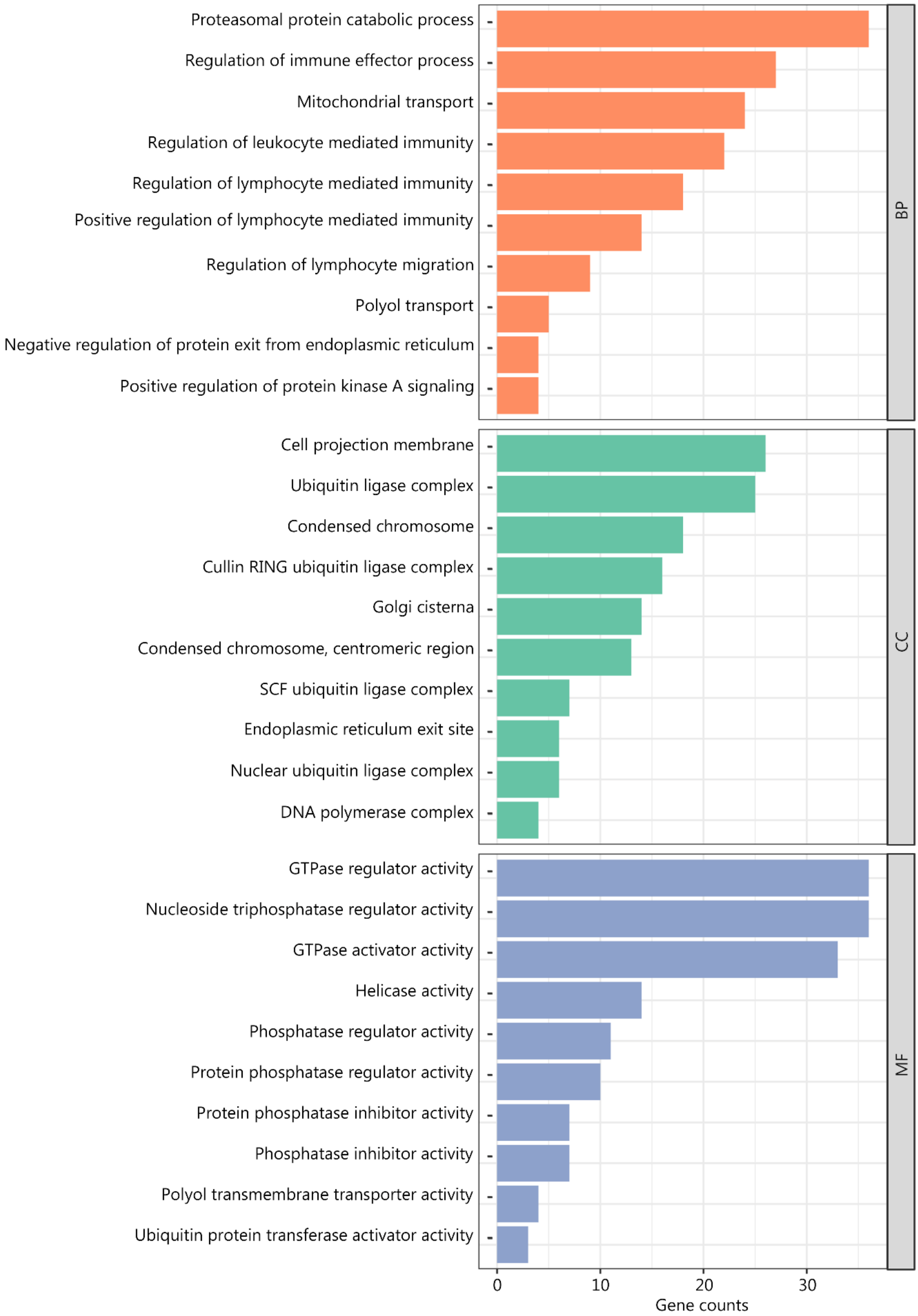

Fig. S9 GO analysis for target genes of eRNAQTL-eRNAs in our CRC tissues. The top 10 significant pathways among BP, MF, and CC are visualized. GO Gene Ontology, BP biological process, MF molecular function, CC cell component, SCF SKP1-CUL1-F-box

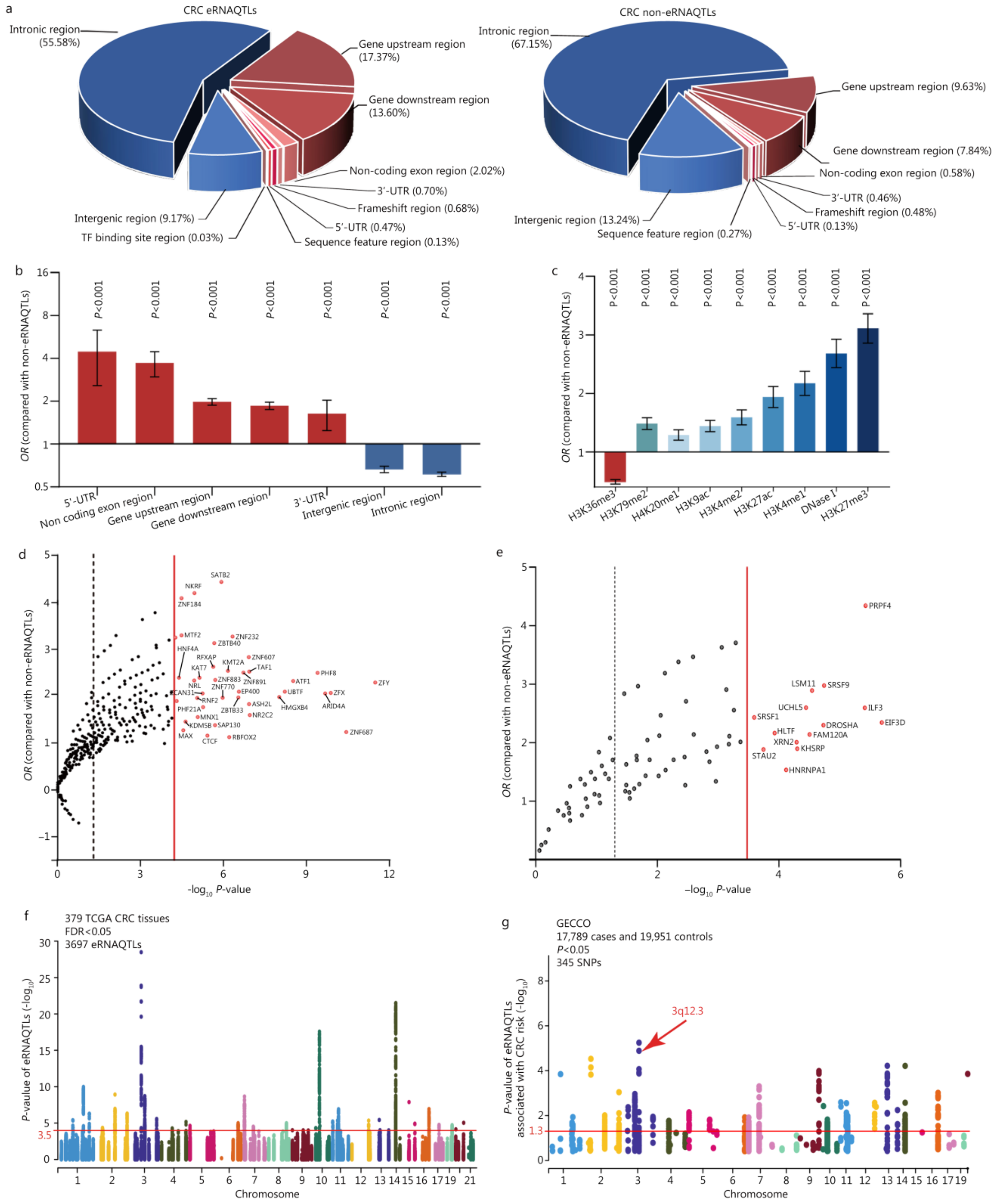

Fig. S10 Genomic and functional characterization of identified eRNAQTLs in colorectal cancer from TCGA dataset. **a** Pie charts describe proportions of SNPs with eRNAQTL or without eRNAQTL fall in various genomic locations. **b** Enrichment analysis of eRNAQTL SNPs in different genomic categories. *P*-values were calculated by a two-tailed Fisher's exact test. Bars indicate 95% confidence intervals (CIs). **c** Enrichment analysis of eRNAQTLs among variants within regulatory

elements. *P*-values were calculated by a two-tailed Fisher's exact test. Bars indicate 95%CIs. **d** Plots of $-\log_{10}$ *P*-values (X-axis) and $-\log_{10}$ *OR* (Y-axis) were obtained from enrichment analysis of eRNAQTLs among variants within binding sites for each TF. The black dashed line indicates $P = 0.05$ and the red solid line indicates $P = 0.05/801$ (6.24×10^{-5}) (Bonferroni-corrected *P*-value threshold, binding sites for a total of 801 TF were tested). **e** Enrichment of eRNAQTL SNPs at the binding sites of individual RBPs using eCLIP-seq data from ENCODE by two-tailed Fisher's exact test. The black dashed line indicates $P = 0.05$ and the red solid line indicates the Bonferroni-corrected *P*-value threshold according to the number of RBPs tested (150 RBPs). **f** Manhattan plot of eRNAQTL results from associations between SNP genotypes and eRNA expression in human CRC tissues from TCGA data. The $-\log_{10}$ *P*-values of the eRNAQTLs (Y-axis) are presented according to their chromosomal positions (X-axis, NCBI build 37). FDR < 0.05 was considered statistically significant (denoted by red line). **g** Manhattan plot for associations between eRNAQTLs and CRC risk in European populations from GECCO. $P < 0.05$ was considered statistically significant (denoted by the red line). TF transcription factor, *OR* odds ratio, TCGA The Cancer Genome Atlas, CRC colorectal cancer, GECCO Genetics and Epidemiology of Colorectal Cancer Consortium, RBP RNA binding protein, FDR false discovery rate, eRNAQTLs eRNA quantitative trait loci, eCLIP-seq enhanced cross-linking immunoprecipitation sequencing, ENCODE Encyclopedia of DNA Elements, SNP single nucleotide polymorphism, FDR false discovery rate

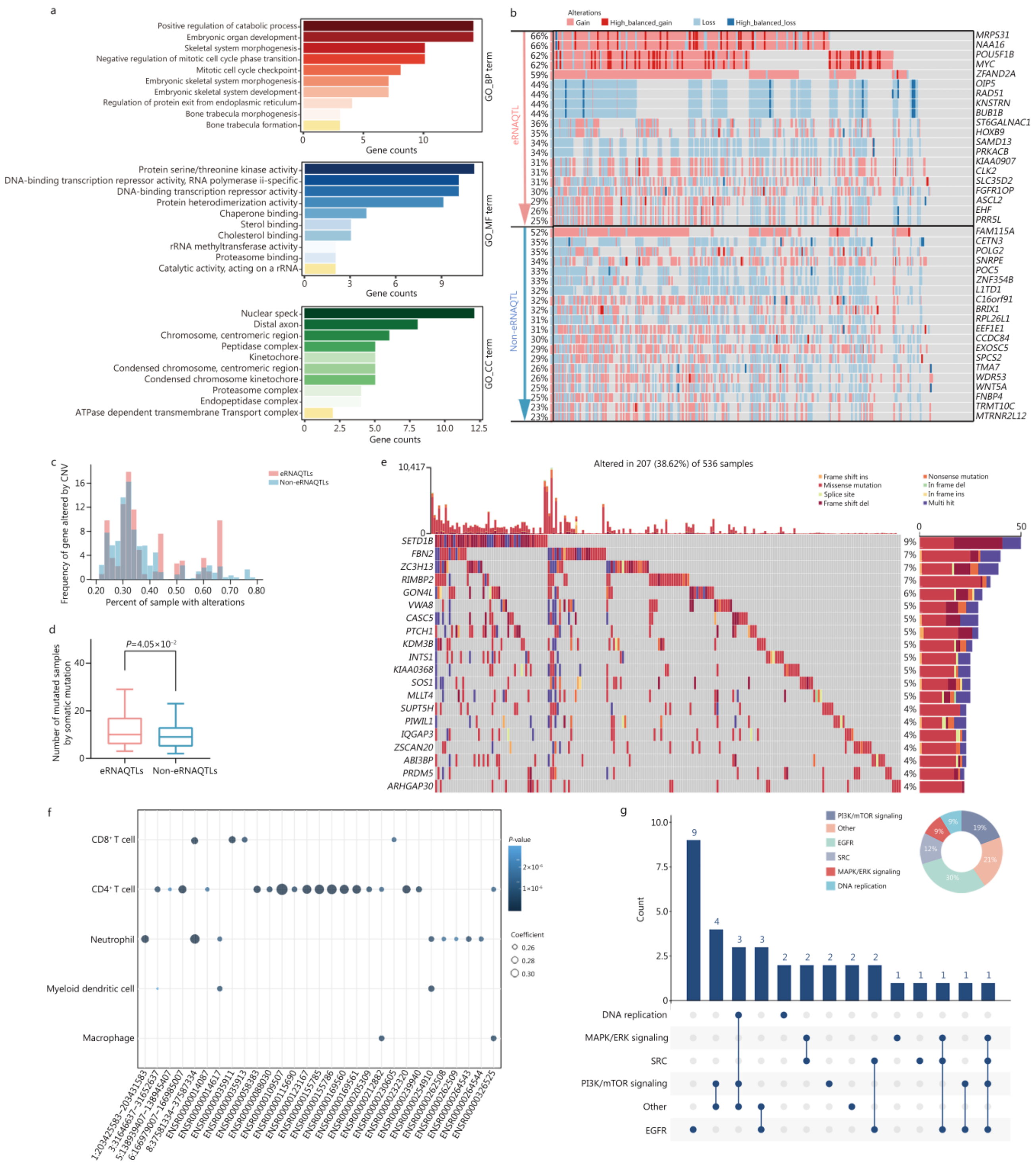

Fig. S11 Characterization of eRNAQTL-eRNAs and putative target genes in colorectal cancer from TCGA dataset. **a** GO analysis for target genes of eRNAQTL-eRNAs in CRC, the top 10 significant pathways among BP, MF, and CC are visualized. **b** Significant copy-number alterations identified by GISTIC2.0. Only the top 20 genes with the highest alteration frequency are displayed. **c** The proportion of TCGA CRC samples have copy-number variation in eRNAs-target genes. **d** Number of eRNAs-target genes affected by somatic mutation. The *P*-value was calculated by a two-sided

Student's *t*-test. **e** Mutation landscape for target genes of eRNAQTL-eRNAs among TCGA CRC samples. The top panel shows individual tumor mutation rates. The right panel shows the frequency distribution of genes affected by different mutation types. Mutation types are indicated in the top legend. **f** Correlations between eRNAQTL-eRNAs (X-axis) and immune cell fractions (Y-axis) were calculated by partial correlation coefficient with tumor purity adjusted. Dot size denotes the magnitude of correlation. **g** Number of eRNAQTL-eRNAs associated with drug response in 6 pharmaceutical targets. The doughnut chart represents the proportion of eRNA-drug pairs in each cancer signaling pathway. GO Gene Ontology, BP biological process, MF molecular function, CC cell component, CNV copy number variation, PI3K/mTOR phosphatidylinositol 3-kinase/mammalian target of rapamycin, EGFR epidermal growth factor receptor, SRC sarcoma proto-oncogene tyrosine-protein kinase, MAPK/ERK mitogen-activated protein kinase/extracellular signal-regulated kinase, eRNAQTL eRNA quantitative trait locus

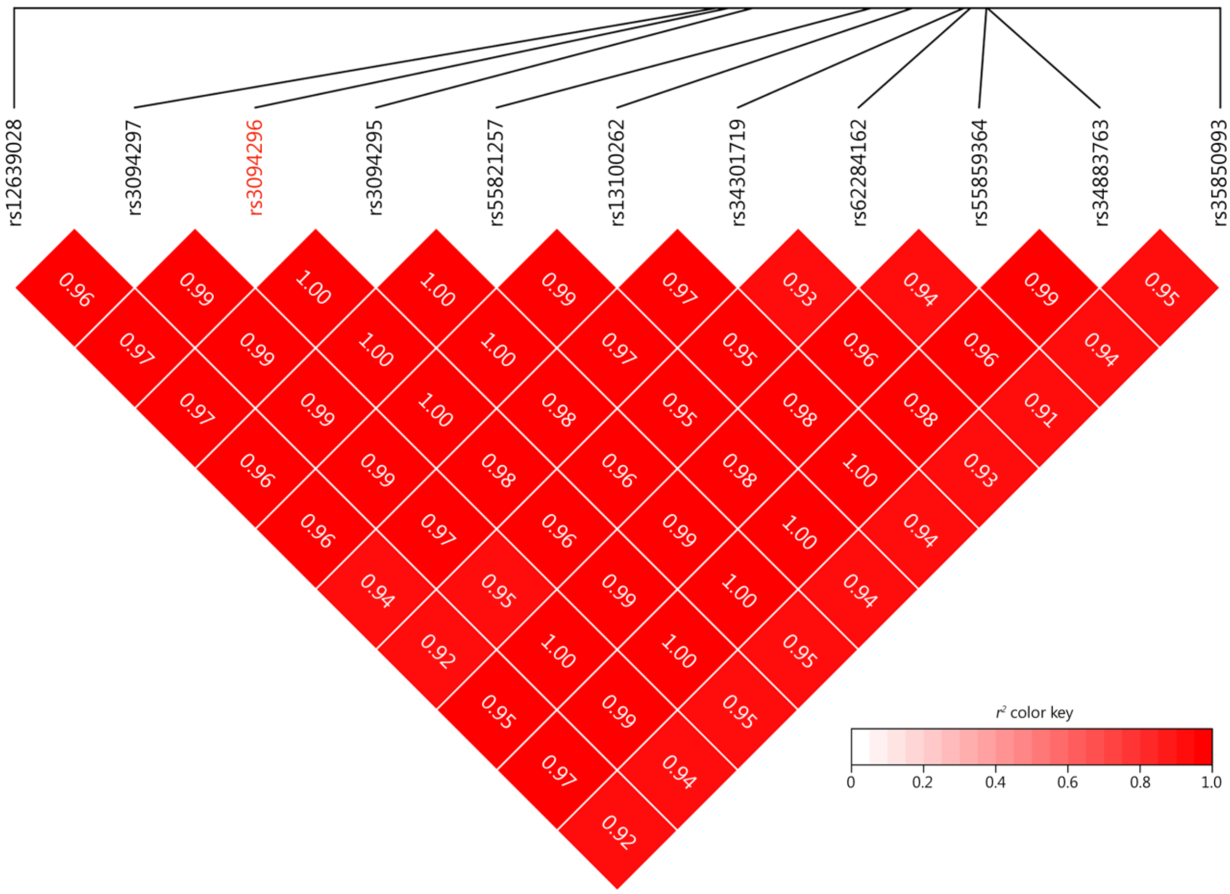

Fig. S12 LD block plot ($r^2 \geq 0.8$) showing the r^2 values of candidate variants. The r^2 values between variants were estimated using the 1000 Genomes June 2014 EUR samples. The most potentially functional variant in this block was selected for further population and experimental validation and labeled in red. LD linkage disequilibrium, EUR European

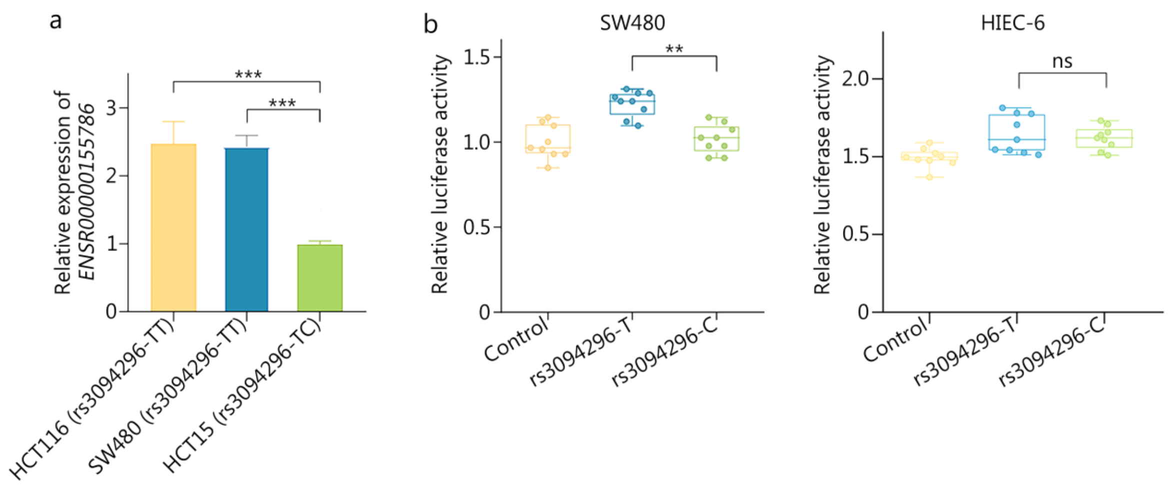

Fig. S13 Relative expression of *ENSR00000155786* in three cell lines. **a** Relative expression of *ENSR00000155786* in HCT116 (rs3094296-TT) and SW480 (rs3094296-TT) cell lines compared to HCT15 (rs3094296-TC) cell line. **b** Relative reporter gene activity of the vectors containing the rs3094296-T or rs3094296-C allele in SW480 and HIEC-6 cell lines. $^{**}P < 0.01$, $^{***}P < 0.001$. ns non-significant

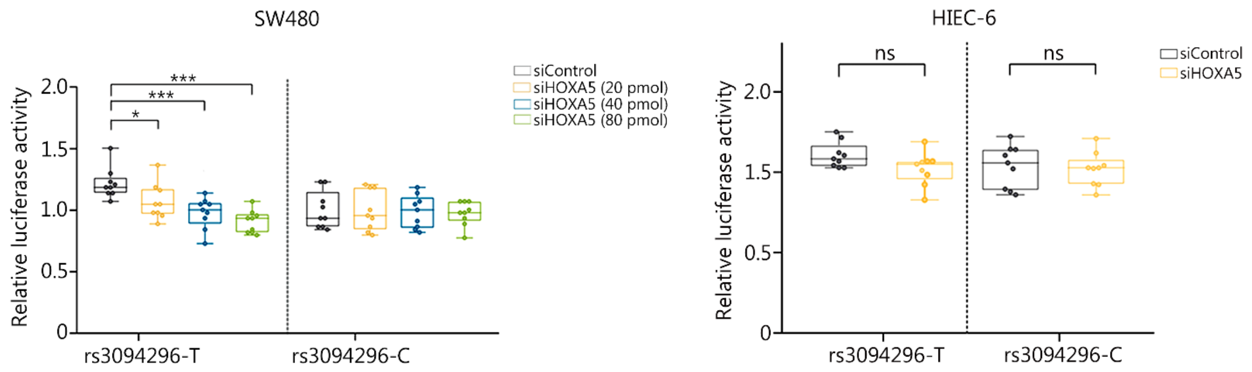

Fig. S14 Effect of *HOXA5* knockdown on the relative luciferase activity of vectors containing the rs3094296-T or rs3094296-C allele in SW480 (left) and HIEC-6 (right) cell lines. Data were presented as the median (minimum to maximum) from three repeated experiments, each has three technical replicates. *P*-values were calculated by a two-sided Student's *t*-test. $^{*}P < 0.05$, $^{***}P < 0.001$. ns non-significant, HOXA5 homeobox A5

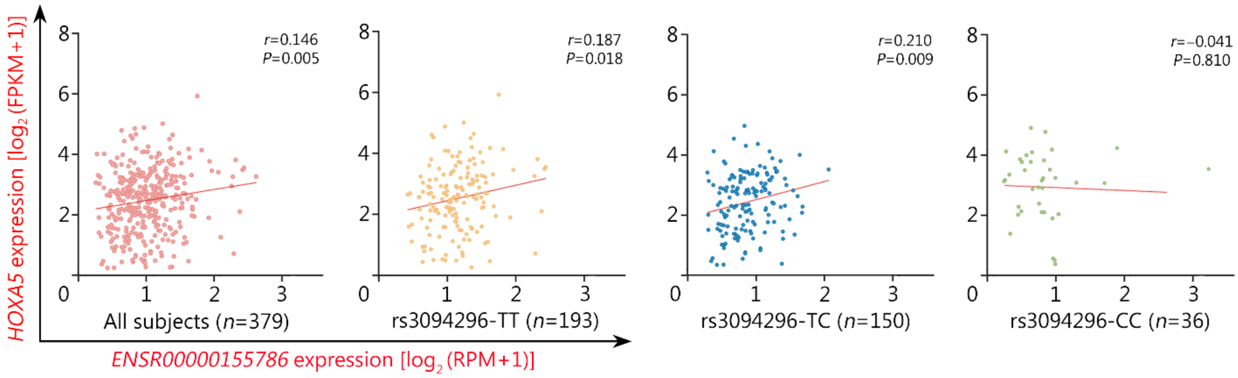

Fig. S15 The correlations between *ENSR00000155786* and *HOXA5* expression in all TCGA CRC samples stratified by SNP rs3094296 genotype. All *P*-values and correlation coefficients were calculated by Spearman's correlation analysis. HOXA5 homeobox A5, TCGA The Cancer Genome Atlas, CRC colorectal cancer, SNP single nucleotide polymorphism, FPKM fragments per kilobase of exon model per million mapped fragments, RPM reads per million

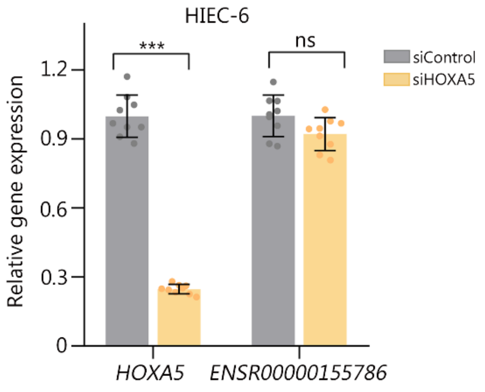

Fig. S16 The effect of *HOXA5* knockdown on the expression level of *ENSR00000155786* in HIEC-6 cells. $^{***}P < 0.001$. ns non-significant, HOXA5 homeobox A5

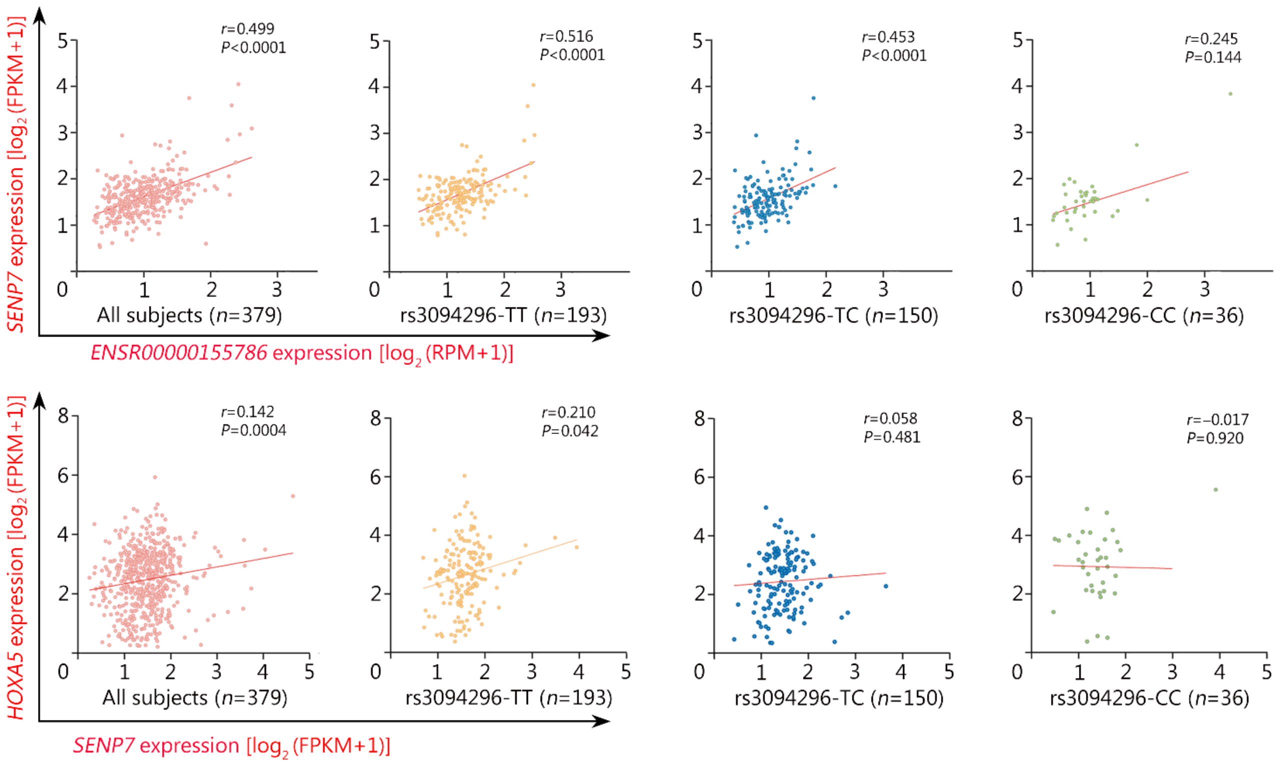

Fig. S17 Correlations of *SENP7* expression with *ENSR00000155786* and *HOXA5* expression in TCGA CRC samples. Scatter plots show the correlations between *ENSR00000155786* and *SENP7* expression (top), as well as the correlations between *SENP7* expression with *HOXA5* expression (bottom) in all TCGA CRC samples, both of them were stratified by SNP rs3094296 genotype. All *P*-values and correlation coefficients were calculated by Spearman's correlation analysis. TCGA The Cancer Genome Atlas, CRC colorectal cancer, FPKM fragments per kilobase of exon model per million mapped fragments, RPM reads per million, SENP7 sentrin-specific protease 7, HOXA5 homeobox A5

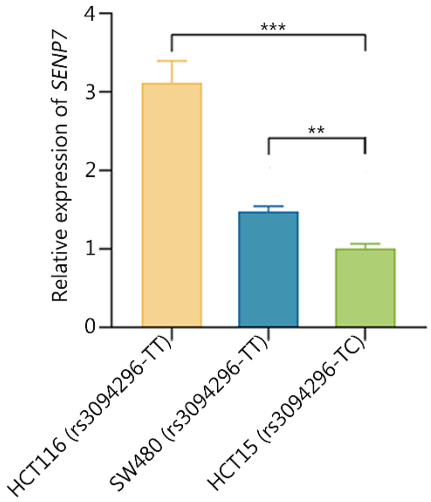

Fig. S18 Relative expression of *SENP7* in HCT116 (rs3094296-TT) and SW480 (rs3094296-TT) cell lines compared to HCT15 (rs3094296-TC) cell line. *P*-values were calculated using a two-sided Student's *t*-test. $^{**}P < 0.01$, $^{***}P < 0.001$. SENP7 sentrin-specific protease 7

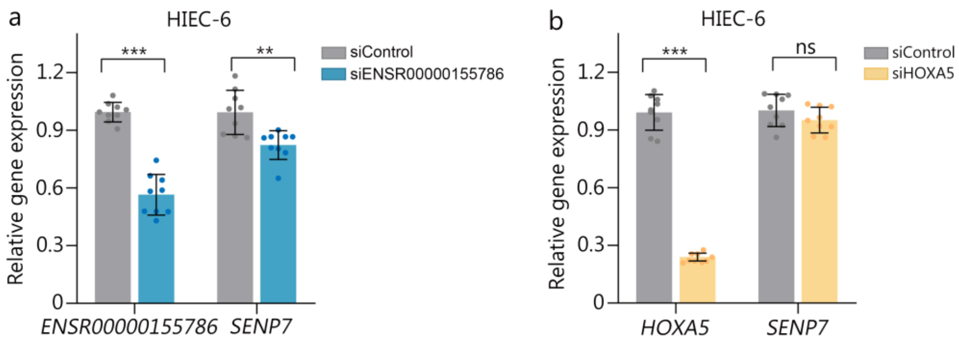

Fig. S19 The expression level of *SENP7* and *ENSR00000155786* in HIEC-6 cells. **a** The effect of *ENSR00000155786* knockdown on the expression level of *SENP7* in HIEC-6 cells. **b** The effect of *HOXA5* knockdown on the expression level of *SENP7* in HIEC-6 cells. $^{**}P < 0.01$, $^{***}P < 0.001$. ns non-significant, SENP7 sentrin-specific protease 7, HOXA5 homeobox A5

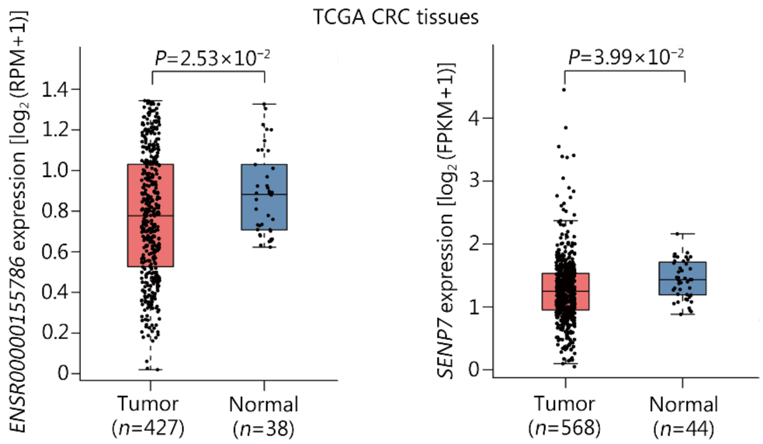

Fig. S20 Differential expression of *ENSR00000155786* and *SENP7* in TCGA CRC samples. *ENSR00000155786* and *SENP7* are significantly decreased in tumor tissues compared with normal tissues from TCGA CRC samples. Data were presented as the median (minimum to maximum). *P*-values were calculated by a two-sided Student's *t*-test. TCGA The Cancer Genome Atlas, CRC colorectal cancer, RPM reads per million, SENP7 sentrin-specific protease 7

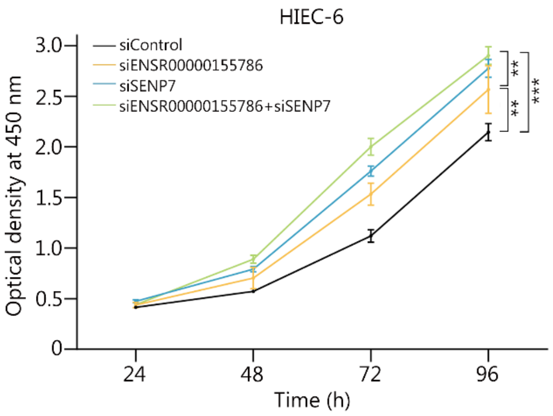

Fig. S21 Cell proliferation assay with knockdown of *ENSR00000155786* and *SENP7* in HIEC-6 cells. $^{**}P < 0.0$, $^{***}P < 0.001$. Results were shown as the means ± SEM from three experiments, each with six replicates. *P*-values were calculated from a two-sided Student's *t*-test by comparing with controls in 96 h. SENP7 sentrin-specific protease 7, SEM standard error of the mean
